# Ecological niches and assembly dynamics of diverse microbial consortia in the gastrointestine of goat kids

**DOI:** 10.1093/ismejo/wrae002

**Published:** 2024-01-11

**Authors:** Jinzhen Jiao, Jian Wu, Chuanshe Zhou, Zhixiong He, Zhiliang Tan, Min Wang

**Affiliations:** CAS Key Laboratory of Agroecological Processes in Subtropical Region, Institute of Subtropical Agriculture, The Chinese Academy of Sciences, Changsha, Hunan 410125, P. R. China; CAS Key Laboratory of Agroecological Processes in Subtropical Region, Institute of Subtropical Agriculture, The Chinese Academy of Sciences, Changsha, Hunan 410125, P. R. China; CAS Key Laboratory of Agroecological Processes in Subtropical Region, Institute of Subtropical Agriculture, The Chinese Academy of Sciences, Changsha, Hunan 410125, P. R. China; University of Chinese Academy of Sciences, Beijing, 101408, China; CAS Key Laboratory of Agroecological Processes in Subtropical Region, Institute of Subtropical Agriculture, The Chinese Academy of Sciences, Changsha, Hunan 410125, P. R. China; University of Chinese Academy of Sciences, Beijing, 101408, China; CAS Key Laboratory of Agroecological Processes in Subtropical Region, Institute of Subtropical Agriculture, The Chinese Academy of Sciences, Changsha, Hunan 410125, P. R. China; University of Chinese Academy of Sciences, Beijing, 101408, China; CAS Key Laboratory of Agroecological Processes in Subtropical Region, Institute of Subtropical Agriculture, The Chinese Academy of Sciences, Changsha, Hunan 410125, P. R. China; University of Chinese Academy of Sciences, Beijing, 101408, China

**Keywords:** gastrointestinal microbiome, goat kids, regional heterogeneity, developmental trajectory, phytobiotics

## Abstract

Goats are globally invaluable ruminants that balance food security and environmental impacts, and their commensal microbiome residing in the gastrointestinal tract (GIT) is associated with animal health and productivity. However, the reference genomes and functional repertoires of GIT microbes in goat kids have not been fully elucidated. Herein, we performed a comprehensive landscape survey of the GIT microbiome of goat kids using metagenomic sequencing and binning, spanning a dense sampling regime covering three gastrointestinal compartments spatially and five developmental ages temporally. We recovered 1002 high-quality metagenome-assembled genomes (termed the goat kid GIT microbial catalog [GKGMC]), 618 of which were novel. They encode more than 2.3 million nonredundant proteins, and represent a variety of carbohydrate-degrading enzymes and metabolic gene clusters. The GKGMC-enriched microbial taxa, particularly *Sodaliphilus*, expanded the microbial tree of life in goat kids. Using this GKGMC, we first deciphered the prevalence of fiber-degrading bacteria for carbohydrate decomposition in the rumen and colon, while the ileal microbiota specialized in the uptake and conversion of simple sugars. Moreover, GIT microorganisms were rapidly assembled after birth, and their carbohydrate metabolic adaptation occurred in three phases of progression. Finally, phytobiotics modified the metabolic cascades of the ileal microbiome, underpinned by the enrichment of *Sharpea azabuensis* and *Olsenella* spp. implicated in lactate formation and utilization. This GKGMC reference provides novel insights into the early-life microbial developmental dynamics in distinct compartments, and offers expanded resources for GIT microbiota-related research in goat kids.

## Introduction

Ruminant livestock possess the capacity to convert human inedible plant biomass into nutritive products such as milk and meat [1], contributing to 45% of global animal protein production [2]. Global drivers such as growing human population, increased urbanization, and environmental sustainability are challenging ruminant livestock unprecedentedly to produce more high-quality products with fewer resources [3]. Goats represent an important ruminant species, and are reared primarily for meat production. A worldwide estimated inventory of one billion goats produced six million tons of chevon in 2020 [4]. Over the past decade, goat meat has gained increased popularity from consumers owing to its functional components, including health-enhancing omega-3 polyunsaturated fatty acids, bioactive phospholipids, B vitamins, iron, and zinc [5]. Therefore, understanding how goats convert plant feed into energy, and subsequently into the functional components of meat, is of vital significance.

Ruminants have evolved a multi-chambered stomach that is specialized in digesting fibrous components into essential metabolic precursors such as short chain fatty acids (SCFAs) and microbial proteins through microbial-mediated fermentation in the rumen [1]. In this regard, the composition, ecology and metabolism of rumen microbial consortia have been predominantly determined through both culture- and omics-based approaches [1, 6, 7]. The contributions of these microorganisms to host physiology, productivity, health, and even the environment have been further explored in dairy cows [8, 9], beef cattle [10] and sheep [11]. The ruminant digestive tract is partitioned into three distinct segments: the stomach (rumen, reticulum, omasum, abomasum), small intestine (duodenum, jejunum, ileum) and large intestine (cecum, colon, rectum) [12]. Each site is spatially specialized with distinct epithelial structures, substrate availabilities, chemical gradients, as well as biogeographically stratified microbiota, and this spatial heterogeneity has been implicated in pronounced phenotypes related to animal productivity and health [13]. To date, the microbial ecology of the small and large intestines of goats has been largely overlooked compared with that of their rumen counterparts.

Advances in sequencing technology and computational methods have spearheaded a revolution in mammalian microbiome research, with particular interest given to microbial origin, metabolism and function. A recent sequencing-based study revealed the presence of a microbiome in the gut of fetal lambs [14]. However, a myriad of researches have asserted that microbial succession within the GIT commences at birth, and is thereafter progressively populated with diverse microbial species in a longitudinal manner before reaching adult complexity [15-18]. The development of the goat kids progressed in three stages starting with an undeveloped nonrumination stage (0–3 weeks), through a transition stage (3–8 weeks) and then to a functionally mature rumination stage (8 weeks onwards) [17, 19]. Increasing evidence has emphasized the materiality of early-life microbiome in the regulation of immune, endocrine, and metabolic developmental pathways. The high plasticity and adaptability of the early-life microbiome underscore the unique window of opportunity for developing strategies to improve long-term health and well-being through modulating them in early life [20]. Despite these findings, the temporal and spatial assemblage dynamics by which the GIT microbiome develops after infancy toward an adult microbiota is still poorly characterized in goats.

Microbial reference genomes are essential resources for understanding the functional role of individual microorganisms and identifying novel microbial lineages [21]. The Hungate 1000 project was a pioneering initiative to enrich the knowledge of rumen microbiome using a culture-based approach, and the genome catalog contained 480 bacterial strains and 21 archaeal strains [6]. Complementally, metagenome binning can capture substantial microbial diversity through direct analysis of genetic information, and subsequent generation of metagenome-assembled genomes (MAGs) [21]. Discoveries enabled by this technology in ruminants have focused on MAGs recovered from rumen microbial consortia from dairy cows and beef cattle [7, 22, 23]. Recent efforts have attempted to recover MAGs from the GIT bacterial consortia of adult goats [12, 24-26]. However, these microbial genomes do not represent the ecosystem diversity in three distinct GIT regions across various developmental phases of goat kids.

To fill this gap, a comprehensive survey of the GIT microbial ecology from a large goat kid cohort was performed using deep metagenomic binning. In total, 124 content samples were collected, spanning three GIT segments (rumen for stomach, ileum for small intestine, and colon for large intestine) sampled over the timespan of five ages (Day 1, 10, 25, 30, and 90 postnatal). We recovered 1002 high-quality MAGs, 618 of which were novel species, and annotated 2 323 457 nonredundant proteins therefrom. These taxonomic and functional repertoires expanded the genetic architecture of GIT microbial resources currently available to goat kids. The GIT biogeography and assembly dynamics of the microbial consortia were further deciphered, highlighting the significance of taking them into account when manipulating the GIT microbiota to enhance goat productivity and wellbeing.

## Materials and methods

### Experimental design and sampling scheme

**Figure 1:**
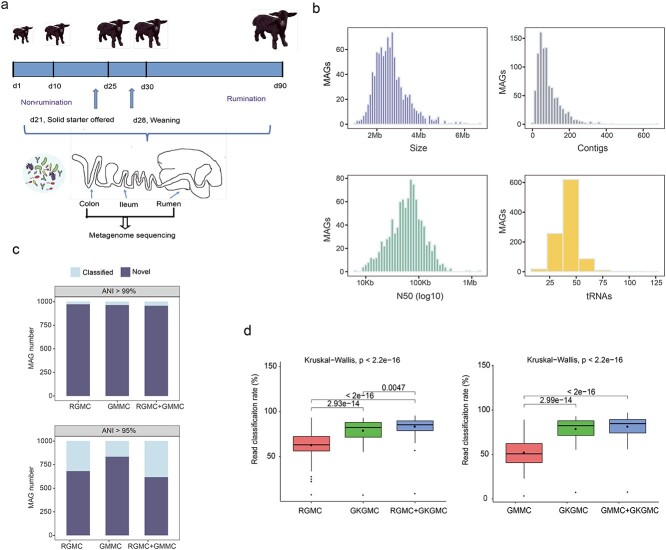
**Recovery of metagenome-assembled genomes (MAGs) from the gastrointestinal microbiome of goat kids. a**, graphical representation of samples spanning three GIT regions and five developmental ages. **b**, histograms showing the distributions of genome sizes, N50 values, numbers of contigs and tRNAs per genome, respectively. **c**, percentages of novel MAGs in goat kid-associated GKGMC (goat kid gastrointestinal microbial catalog) as compared with ruminant-associated RGMC (ruminant gastrointestinal microbial catalog) and goat-associated GMMC (goat multi-kingdom microbial catalog) datasets at 95 and 99% average nucleotide identity (ANI). **d**, read classification rate of 124 samples using our GKGMC in comparison to ruminant-associated RGMC or goat-associated GMMC datasets.

All the experimental procedures were approved by the Animal Care Committee, Institute of Subtropical Agriculture, The Chinese Academy of Sciences, Changsha, Hunan Province, China (permission No. CAS2019020). Sixty new-born Xiangdong black goat kids (*Capra hircus*, a native breed in Hunan Province) with an initial weight of 2.1 ± 0.2 kg were selected as experimental animals (Additional file 3: Table S1), and lived with their dams while breast feeding [27]. All the goat kids were provided with a solid feed with concentration and alfalfa at a ratio of 70:30 at d21, and they imitated their mother’s behavior to eat solid feed. Subsequently, goat kids were weaned at d28, transferred to a well-ventilated room individually, and fed *ad libitum* with solid feed. Twelve goat kids were slaughtered at d1, d10 (nonrumination), d25, d30 (transition) and d90 (rumination) after birth, respectively. The GIT was immediately tied off separately, and luminal contents from the rumen, ileum and colon (Fig. 1a) were homogenized, and thereafter frozen in liquid nitrogen prior to microbial DNA extraction. Profiling of SCFAs in the GIT contents and histomorphological analysis of GIT tissue (Additional file 1, Additional file 2: Fig. S1) were conducted as described previously [19].

### Metagenomic sequencing, assembly, and binning

Microbial DNA extraction was conducted via the bead-beating method as detailed previously [28]. The DNA yield and integrity were assessed using a Qubit 2.0 Fluorometer. Metagenomic libraries with an insert of 450 bp were constructed with TrueSeq DNA PCR-Free Library Prep Kits following the manufacturer’s instructions. The samples were sequenced on the 150 bp paired-end Illumina NovaSeq or BGISEQ platform (Additional file 3: Tables S2–S3).

Raw data were quality filtered using Trimmomatic (v.0.35) [29]. Reads aligned to the reference goat genome (ARS1, GCA_001704415.1) using BWA-MEM [30] were removed, and 69.6% of the reads were retained as clean reads; these were subsequently *de novo* assembled separately using MEGAHIT [31]. Contigs longer than 1.5 kb were selected for binning into MAGs using metaBAT2 with default parameters [32]. The completeness and contamination of the MAGs were estimated with CheckM [33]. Genome quality was defined as completeness-5 × contamination, and only MAGs with a quality of ≥50 were retained [34]. High-quality MAGs were refined according to the thresholds of ≥90% completeness and ≤ 5% contamination [34], and then dereplicated with a 99% ANI cutoff using dRep [35]. Consequently, 1002 nonredundant high-quality MAGs (Additional file 3: Table S4) were obtained, and referred to as GKGMC.

### Comparisons with public ruminant- or goat- GIT reference microbial genomes

To extend the GIT microbial genomes of goat kids into a comprehensive ruminant-associated or goat-associated microbial tree of life, two large-scale datasets were compiled and generated. The ruminant GIT microbial catalog (RGMC) comprises of seven publicly available large cohort studies in ruminants and Hungate collection genomes (20.9 Tbp metagenomic data, access to October 2022; Additional file 2: Fig. S2, Additional file 3: Table S5). The goat multi-kingdom microbial catalog (GMMC) was recently published and consisted of 4004 bacterial and 71 archaeal genomes [25]. Either catalog was extracted with ≥90% completeness and ≤ 5% contamination, followed by dereplication at 99% ANI using dRep [35]. As a result, the RGMC (ruminant-associated) and GMMC (goat-associated) datasets included 6260 and 2121 microbial genomes, respectively. Thereafter, our goat kid-specific GKGMC was aligned with RGMC and GMMC using FastANI, according to previously reported genome thresholds for species and strain delineation of ≥95% and ≥ 99% ANI, respectively, and ≥ 60% of the genome [36]. Those MAGs that did not match any available references were referred to as novel genomes.

### Taxonomic and functional analysis of GKGMC

The MAGs were taxonomically annotated using GTDB-Tk (v1.7.0) [37]. Phylogenetic tree was generated using PhyloPhlAn [38], and visualized in GraPhlAn [39]. The gutSMASH tool [40] was used to identify metabolic gene clusters (MGCs). Subsequently, GKGMC-encoded proteins were predicted using METAProdigal [41], followed by clustering into nonredundant sequences using CD-HIT [42]. Amino acid sequences were aligned to the KEGG database using KofamKOALA [43], and also searched against eggNOG with eggNOG-mapper [44], and against CAZyme with the hmmscan program in HMMER [45]. Prediction of polysaccharide utilization loci (PUL) was carried out following the protocol of PULDB [46]. These sequences were annotated against the HydDB database to identify the hydrogenases responsible for hydrogen production and consumption [47].

### Estimation of the abundances of MAGs, MAG-encoded genes, and functional terms

Clean reads were aligned to the contig set of GKGMC using BWA-MEM [30] to calculate the read depth of MAGs. After converting the resulting SAM files to BAM format using SAMtools, Salmon [48] was employed to calculate the abundance of each MAG based on their respective contig read depth in each sample. In parallel, the clean reads were aligned to the GKGMC-encoded gene catalog using BWA-MEM [30]. After converting format with SAMtools, Salmon [48] was applied to quantify the number of successfully assigned reads. The gene abundance profiles in each sample were normalized to transcripts per million (TPM) values, with corrections for variations in gene length and mapped reads per sample [49]. The relative abundances of KOs, CAZymes, and hydrogenases in each sample were calculated from the abundances of GKGMC-encoded genes by adding the abundances of all the members falling within each category [50].

### Statistical analysis

Analyses of the alpha and beta diversities of taxonomic and functional profiles of the GIT microbiome were performed in R using the vegan package. Principal coordinate analysis (PCoA) was performed using bray–curtis dissimilarity matrix. After homogeneity of variances was tested with betadisper, the significance of grouping in the PCoA plots was assessed applying the adonis test with 999 permutations. Furthermore, comparisons of microbial taxonomies, genes, KOs, and CAZymes among different GIT regions were performed using the Kruskal-Wallis test, and a false discovery rate (*fdr*) < 0.05 was set as the significance threshold. Concurrently, the taxonomic and functional abundances of microorganisms in each individual GIT region were subjected to pairwise comparisons (d10 vs. d1; d25 vs. d10; d30 vs. d25; d90 vs. d30) to explore dynamic changes during goat development. The Wilcoxon rank-sum test was applied, with significant differences indicated by a *fdr* < 0.05.

### Analysis of the gut microbiome in response to prebiotics in goat kids

Using GKGMC as a reference, we assigned ileal metagenomic data from weaning Xiangdong black goat kids fed a control, antibiotics (control diet plus 21 mg/kg/d vancomycin and 42 mg/kg/d neomycin) or phytobiotics (control diet plus 0.3 g/day *Macleaya cordata* extract) diet in our parallel study [51]. The BWA-MEM [30] was applied to assign taxonomic and functional levels. The PCoA was performed based on the taxonomic and functional profiles, followed by the betadisper and adonis tests. Comparisons of microbial taxonomies and KOs among the three groups were performed using the Kruskal-Wallis test. Differentially enriched MAGs were identified using DESeq2 [52] for the phytobiotics vs. control and antibiotics vs. control comparisons, with a threshold of log_2_ fold change ≥1.5, and *fdr* < 0.05.

## Results

### Identification of novel MAGs improved the classification rate of metagenomic inventories in goat kids

To elucidate the microbial drivers for GIT morphological and functional maturation during goat development (Additional file 1), we employed a deep metagenomic sequencing of genomic DNA from 124 samples across three GIT regions over the time span of five developmental ages in goat kids (Fig. 1a). A total of 1.79 Tb of clean data enabled recovery of 1002 nonredundant high-quality MAGs. The median size was 2.53 Mb, with N50 values ranging from 6.64 Kb to 1.35 Mb. The GKGMC contained a median of 70 contigs and an average of 42 tRNA genes (Fig. 1b). Moreover, we analyzed the novelty of the GKGMC genome by comparison with the ruminant-associated RGMC or goat-associated GMMC dataset, and found that 61.7% (n = 618) and 95.5% (n = 957) of the MAGs were novel at ANI thresholds of 95% and 99%, respectively (Fig. 1c).

We further assessed the improvements by GKGMC for read classification rates of metagenomic datasets using our goat kid sampling cohorts, and found that 79.0% of the reads could be mapped to GKGMC genomes. This rate was 25.7% or 51.3% greater than (p < 0.01) that in the RGMC and GMMC datasets, respectively (Fig. 1d). Although read classification increases varied among GIT regions, it was greater in early life than those in later life (Additional file 2: Fig. S3). These findings underscore the materiality of sampling underrepresented GIT regions at early life, and indicate that the majority of microorganisms in the whole GIT of goat kids at various developmental phases are well represented by GKGMC.

### Taxonomic annotation of GKGMC reveals highly diverse microbial consortia and expands the microbial tree of life in goat kids

**Figure 2:**
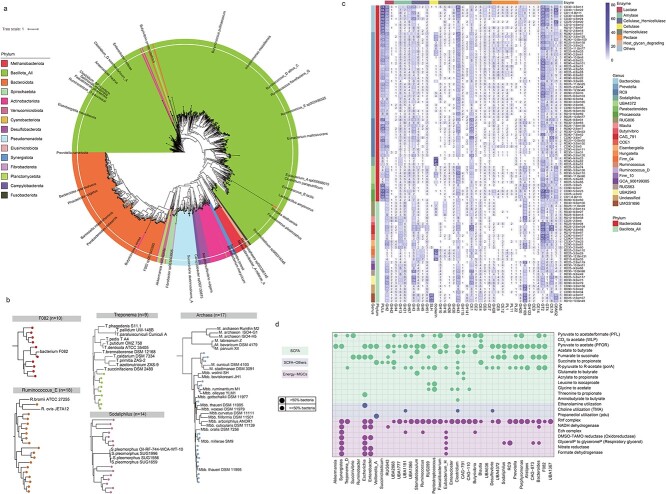
**Taxonomic and functional repertoire of the GKGMC. a**, phylogenetic analysis of 1002 microbial genomes. Labels show prevalent genome names, and are chosen to be informative but not overlapped. **b**. the phylogenetic tree of GKGMC enriched microbial taxa, including F082, *Ruminococcus_E*, *Sodaliphilus*, *Treponema*, and *archaea*. Nodes represent MAGs from the GKGMC, and other homologous strains are retrieved from the NCBI genomes (additional file 1). **c**, Heatmap showing counts of polysaccharide-degrading CAZymes, as well as polysaccharide utilizing loci (PULs) encoded in each MAG. **d**, circles representing the absence/presence of known MGC pathways in each genus. Larger circles indicate cases in which more than 50% of the genomes for a genus encode the pathway, whereas smaller circles indicate cases in which 50% or fewer of the genomes encode it. Colored ranges indicate a categorization of MGCs by chemical class of their product.

Using GTDB-Tk, 985 and 17 MAGs were assigned to bacteria and archaea, respectively. Fourteen and three archaeal MAGs were resolved as *Methanobrevibacter* and *Methanosphaera*, respectively. Among the bacterial MAGs, 377 were resolved to species, 899 to genus, 982 to family, and 985 to order and higher levels (Additional file 3: Table S6). The phylogenetic tree (Fig. 2a) was dominated by two large clusters representing *Bacillota* (n = 651) and *Bacteroidota* (n = 211). Of genomes classified at the species level, we reassembled genomes derived from the strains of *Prevotella ruminicola*, *Ruminococcus flavefaciens*, *Ruminococcus albus, Ruminobacter amylophilus*, *Succinivibrio dextrinosolvens*, *Synergistes jonesii*, *Treponema bryantii*, and *Sharpea azabuensis* (Fig. 2a). These strains are prevalent core rumen members specializing in converting fibrous feed into SCFAs [1]. Moreover, several representatives of segmented filamentous bacteria associated with the modulation of host immune homeostasis [53], including *Bacteroides fragilis*, *Bacteroides thetaiotaomicron*, *Bacteroides uniformis*, *Enterococcus faecalis*, *Ruminococcus gnavus,* and *Parabacteroides distasonis*, were also identified. One MAG was predicted to be *Akkermansia muciniphila*, which supported maintenance of GIT barrier integrity by enhancing mucus production.

Furthermore, we compared the GIT microbial compositions among ruminant-associated (RGMC), two goat-associated datasets (GMMC, and goat fecal microbial catalog (GFMC) from Zhang et al., (2022) [26]), and goat kid-associated GKGMC in this study. The phylum and genus clades were remarkably differed in proportions among the four datasets (Additional file 2: Fig. S4–S5, Additional file 3: Tables S7–S8), and these differences can by caused by genetics, geography, age, GIT region, and diet specificity of ruminants. Strikingly, GKGMC was enriched for several taxa, with particular interest in the underrepresented F082, *Ruminococcus_E*, *Sodaliphilus*, *Archaea*, and *Treponema*. Further phylogenetic analysis of these taxa (Additional file 1, Additional file 3: Tables S9) demonstrated that they uniformly presented a distant evolutionary distance from known members, and might be an evolutionary repertory of these species (Fig. 2b). These findings greatly expanded the microbial tree of life in goat kids.

### Functional repertoire of GKGMC indicates a variety of carbohydrate-degrading enzymes and metabolic gene clusters

We predicted 2 323 457 nonredundant genes from GKGMC, of which 584 850 (25.2%) and 1 825 491 (78.6%) genes could be functionally annotated using KEGG and eggNOG, respectively (Additional file 2: Fig. S6). These genes encoded 2904 KOs and 4314 COGs, a majority of which were involved in the metabolism of carbohydrates, amino acids, energy, cofactors and vitamins, as well as microbial membrane transport, and translation. In contrast, only 5.4% of the genes were annotated as CAZymes, with glycoside hydrolases (GHs) being the predominant family. The CAZyme repertoire encompasses a wide variety of activities in the depolymerization cascade of polysaccharides, associated with functional categories of cellulases, hemicellulases, amylases, and pectinases (Additional file 3: Table S10).

These diversified CAZymes exhibited distinct genus-level distributions (Fig. 2c, Additional file 3: Table S11). Intriguingly, GH5, GH8, GH44 and GH51, which were versatile in hydrolyzing cellulose and hemicellulose, were found in high abundance in MAGs assigned to CAG-791, *Fibrobacter* and *Ruminococcus*, while members of *Sodaliphilus* (GKGMC-enriched taxa as mentioned above) encoded 63 to 107 cellulosome dockerins. Another scenario highlights the importance of members of *Bacteroides*, UBA4372, and *Phocaeicola* in fine-tuning polysaccharide recognition and substrate direction, underpinned by more than ten genes encoding CBM32 in their genomes. Generally, carbohydrate degradation is orchestrated by PULs, and hundreds of gene configuration types of PULs were predicted for breakdown of starch, xylan and pectin in the GKGMC (Additional file 3: Table S12). These delicate coregulated gene sets provide novel insight into how GIT microorganisms sense nutrient availability, and capture, transport, uptake and digestion of glycans. Those MAGs harboring more than 50 PULs were resolved as *Bacteroides*, *Phocaeicola*, *Parabacteroides*, or RC9, while most *Prevotella* MAGs carried approximately 20 PULs (Fig. 2c). Overall, GIT microorganisms orchestrate the depolymerization of dietary polysaccharides through a myriad of CAZymes in a fine-tuning manner.

The GIT microbiota produces numerous bioactive molecules implicated in microbial ecology and host physiology. A total of 2535 MGCs were recovered with a threshold of 70% similarity using gutSMASH, with SCFAs (51.8%) and E-MGCs (26.4%) as the prevalent classes. Remarkable interphylum differences were noted for MGC class distribution (Additional file 2: Fig. S7). In terms of SCFA production, butyrate production was found mainly in members of *Butyrivibrio*, *Faecalibacterium*, *Stomatobaculum*, and UBA2868 through the acetate-to-butyrate pathway (Fig. 2d). Propionate biosynthesis was largely confined to *Veillonella_A*, UBA2868, and *Blautia* through the succinate-to-propionate pathway, and to *Escherichia* through the threonine-to-propionate pathway. Two main acetate production pathways, pyruvate-to- acetate/formate and pyruvate:ferredoxin-oxidoreductase (PFOR), were widely distributed across diverse genera, and other acetate production pathways were confined to only one to four genera. In terms of E-MGCs, *Escherichia*, *Enterobacter*, and *Synergistes* displayed a distinct mechanism of energy capture from others. Specifically, expect for the common Rnf complex for generating proton motive force, they also employed alternative terminal electron acceptors (nitrate, DMSO-TMAO) and NADH dehydrogenase to fuel their metabolism. In addition to the metabolic versatility of *Clostridium* spp. (9 of 17 SCFA pathways), the discovery of some previously unreported taxa such as CAG-110, CAG-791, RC9, RUG099, UBA1066, and UBA2868, manifesting prospect of these underrepresented taxa for SCFA production and host health promotion.

### Gastrointestinal microbiome is constrained by the GIT region and developmental age

**Figure 3:**
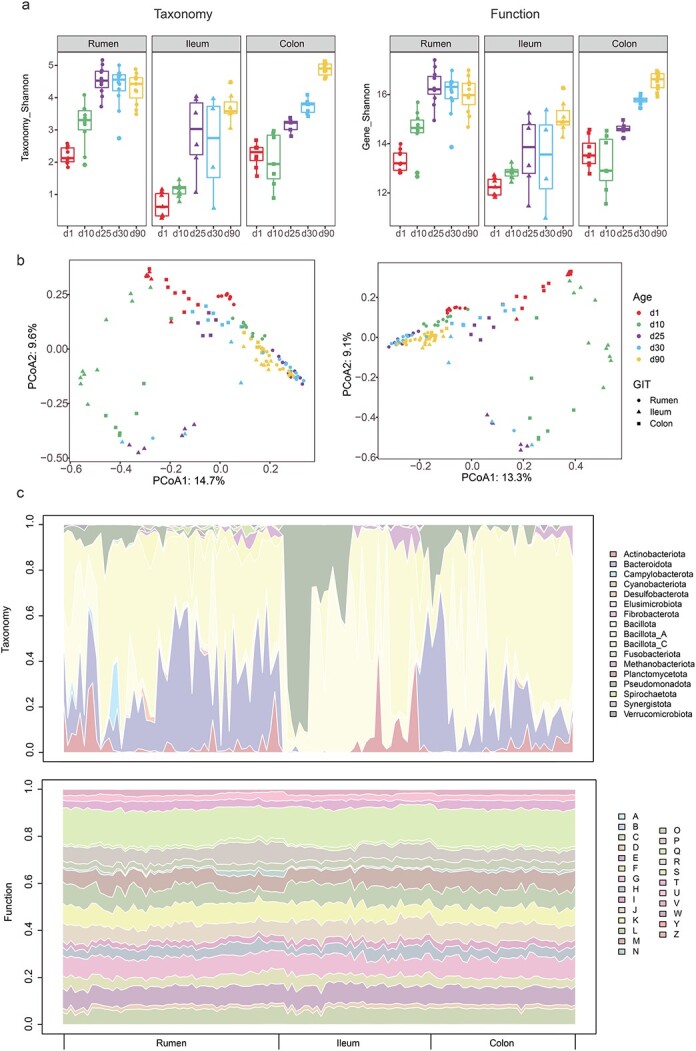
**Taxonomic and functional profiles of gastrointestinal microbiome is constrained by GIT region and developmental age in goat kids. a**, alpha diversity indicated by Shannon at the MAG (taxonomy) and gene (function) levels; **b**, PCoA analysis based on bray–Curtis dissimilarity. The microbial samples from GIT regions were indicated as different shapes, and various developmental ages were indicated by filling colors; **c**, stream graph displaying of phyla and COG functions along the 124 GIT samples. The X-axis indicates the samples clustered by the sampling sites along the GIT. The Y-axis indicates the relative abundances of the phyla and COG functions in each sample. **a**, RNA processing and modification; **b**, chromatin structure and dynamics; **c**, energy production and conversion; **d**, cell cycle control, cell division, chromosome partitioning; **e**, amino acid transport and metabolism; **f**, nucleotide transport and metabolism; **g**, carbohydrate transport and metabolism; **h**, coenzyme transport and metabolism; **i**, lipid transport and metabolism; **j**, translation, ribosomal structure, and biogenesis; **k**, transcription; **l**, replication, recombination and repair; **m**, cell wall/membrane/envelope biogenesis; **n**, cell motility; **o**, posttranslational modification, protein turnover, chaperones; **p**, inorganic ion transport and metabolism; **q**, secondary metabolites biosynthesis, transport, and catabolism; **r**, general function prediction only; **s**, function unknown; **t**, signal transduction mechanisms; **u**, intracellular trafficking, secretion, and vesicular transport; **v**, defense mechanisms; **w**, extracellular structures; **y**, nuclear structure; **z**, cytoskeleton.

Using GKGMC as a reference, we deciphered the GIT biogeography and assembly dynamics of microbial consortia in goat kids. Alpha diversity as measured by the Shannon index at both the taxonomic and genetic levels dramatically increased with developmental ages (*p* < 0.01; Fig. 3a). We also observed a decrease in the Shannon index from the rumen to the ileum, followed by an increase in the Shannon index in the colon. Principal coordinate analysis showed that both GIT region and developmental age exerted pronounced effects on microbial composition (PERMANOVA, F_GIT_ = 10.6, F_Age_ = 4.7, F_GIT_ × F_Age_ interaction = 5.7, *p* < 0.01) and function (PERMANOVA, F_GIT_ = 10.2, F_Age_ = 3.8, F_GIT_ × F_Age_ interaction = 3.4, *p* < 0.01; Fig. 3b). The overall microbial structures of rumen, ileum and colon were different from each other, with *Bacillota* and *Bacteroidota* being the most abundant phyla (Fig. 3c). Synchronously, each GIT region possessed distinct functional potentials, with “carbohydrate transport and metabolism”, “amino acid transport and metabolism”, and “replication, recombination and repair” being the top functions. In summary, the GIT region and developmental age jointly contributed to shaping the microbial composition and functional potential.

### Distinctive regional compositional patterns of MAGs coincide with their functional potentials

**Figure 4:**
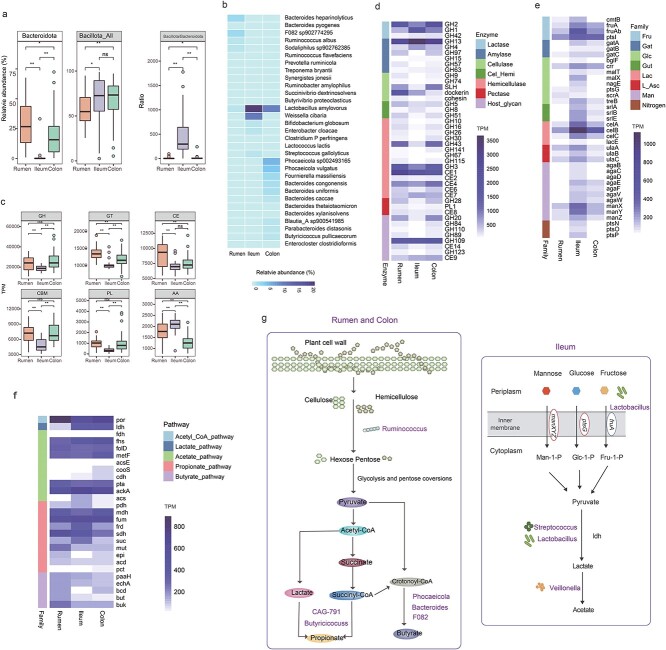
**GIT biogeography of microbial composition and functional potential.** Spatial variation of the relative abundance of taxa of interest in the three GIT regions at phylum (**A**) and species (**B**) levels; comparing the abundance of CAZymes of interest in the three GIT regions at class (**C**) and family (**D**) levels; Heatmaps showing the spatial distribution of major KOs responsible for phosphotransferase system (**E**) and SCFA production (**F**). **G**, graphical scheme of distinct patterns of MAGs correlate with their function within the GIT. Only regional differential taxonomic and functional terms were presented in the heatmaps. Level of significance in the boxplot: Ns *p* ≥ 0.05, ^*^p < 0.05, ^*^^*^p < 0.01.

Regional heterogeneity was noted for both microbial composition and function (Additional file 2: Fig. S8). *Bacteroidota* showed the lowest abundance in the ileum, while the *Bacillota_all* exhibited similar high abundance in the ileum and colon (Fig. 4a). Active fiber- and starch-degraders, including *R. albus*, *R. flavefaciens*, *Sodaliphilus* sp902762385, *P. ruminicola*, *T. bryantii*, *S. jonesii*. *R. amylophilus*, and *S. dextrinosolvens* were dominant in the rumen; *Phocaeicola vulgatus*, *Bacteroides congonensis* and *B. uniformis* were prevalent in the colon; while the lactate producers *Lactobacillus amylovorus* and *Weissella cibaria* exhibited tremendously greater abundances in the ileum (Fig. 4b).

Almost all CAZy families exhibited the highest abundance in the rumen, followed by the colon and then the ileum (Fig. 4c). Notably, the ileum exhibited a greater abundance of lactase GH1 and α-amylase GH13, whereas greater abundances of cellulase SLH and dockerin, β-xylosidase GH43, hemicellulase GH3 and CE1 were observed for the rumen and colon (Fig. 4d). Further insight into SCFA biosynthesis pathways demonstrated that the ileum was enriched for the lactate (*ldh*) and acetate (*ackA*, *acs*) production pathways (Fig. 4f), predominantly phylogenetically derived from *L. amylovorus* and *Escherichia flexneri* (Additional file 4: Tables S13–S14, identified by taxonomy of the MAG harboring the corresponding gene). In contrast, the rumen and colon were enriched for pathways of propionate production via succinate as an intermediate (*sdh*, *epi*, *pct*), together with butyrate production (*buk*). Heterotrophic bacteria rely on the metabolism of organic carbon sources acquired from the environment, and their phosphotransferase system (PTS) is dedicated to the transport and phosphorylation of sugars, such as glucose, lactose, fructose, mannose, and cellobiose [54]. Intriguingly, genes encoding enzymes responsible for PTS systems (Additional file 4: Tables S15–S16), spanning the Fru, Gat, Glc, Gut, Lac, L-Asc, Man, and nitrogen families, showed preciously consistent marginal increases in abundance in the ileum when compared with the rumen and colon (Fig. 4e). Collectively, the colonic microbiota closely resembles the microbiota of the rumen, where complex carbohydrates are fermented, but differs substantially from the ileal microbiota, which specializes in the uptake and conversion of simple mono- and disaccharides (Fig. 4g).

### GIT microorganisms are rapidly assembled after birth, and shifts in microbial functional metabolic plasticity coordinate GIT development

**Figure 5:**
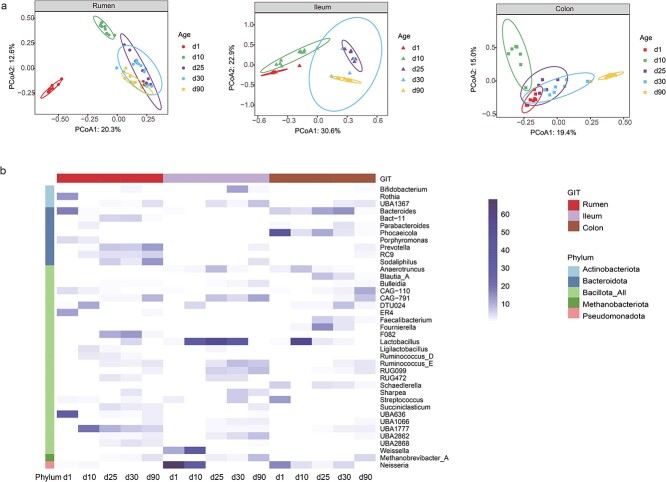
**Microbial composition dynamics in the GIT of goat kids from birth to rumination. A**, PCoA of GIT microbiome at the MAG level based on bray–Curtis dissimilarity in the rumen, ileum and colon, respectively; **B**, relative abundances of microbial genera in each GIT regions for each developmental age. Only differential genera were presented.

To disentangle the GIT region × developmental age interaction effect, we focused on temporal changes within individual GIT regions. Consistent progressive dynamic microbiome patterns were detected independent of GIT regions, as measured by beta diversity analysis (Fig. 5a). *Methanobrevibacter_A* spp. experienced a similar increase with age in all GIT regions, whereas a heterogeneous colonization of bacterial genera was observed in each GIT region (Fig. 5b). In the rumen, UBA636, *Bacteroides*, *Rothia*, and *Porphyromonas* spp. were the dominant members of newborn goats, while their abundances decreased substantially at d10, accompanied by surges in DTU024 and UBA1777. As we progressed with developmental age and dietary transition from milk to a solid diet, we observed an increase in the abundances of *Prevotella*, *Sodaliphilus*, CAG-791, and F082 in the rumen. The newborn ileum was dominated by the immune modifier *Neisseria* (68%). Subsequently, the relative abundances of probiotic-lactate bacteria *Lactobacillus* and *Weissella* increased 42.5- and 2.0-fold during provision of goat milk, but decreased remarkably after weaning to <1% at d90. Concurrently, the prevalence of carbohydrate-degraders CAG-791, RUG099, UBA1367, *Sodaliphilus*, and *Sharpea* was noted for d90 in the ileum. Intriguingly, the colonic microbiota exhibited a temporal colonization shift in colonization from *Phocaeicola* (39.4%), *Neisseria* (15.8%), and *Streptococcus* (12.4%) at d1, to *Lactobacillus* (54.3%) during milk provision, and eventually to GAG-110 (10.2%), and CAG-791 (7.0%) at d90 (Fig. 5b). Furthermore, co-occurrence network identified hub microbes in each GIT region (Additional file 1, Additional file 2: Fig. S9), and analysis of microbial community assembly processes indicated that their assembly was orchestrated by fine-scale deterministic and stochastic ecological processes in the GIT of goat kids (Additional file 1, Additional file 2: Fig. S10).

**Figure 6:**
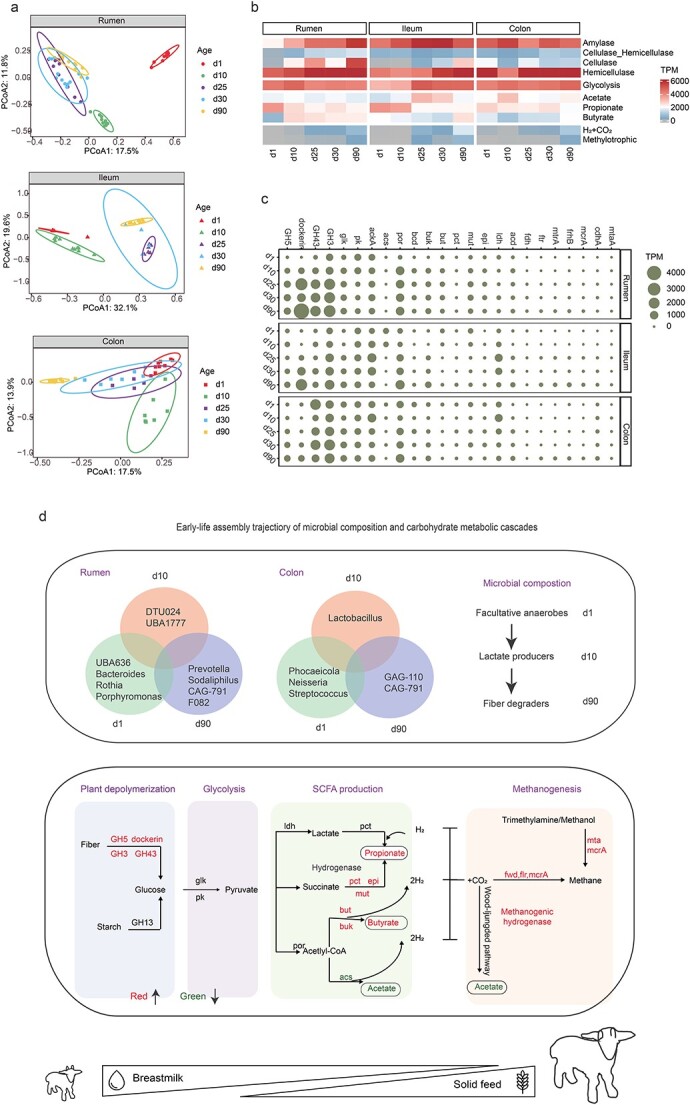
**Microbial functional maturation in the GIT microbiota of goat kids from birth to rumination. A**, PCoA of GIT functional potentials at the gene level based on bray–Curtis dissimilarity in the rumen, ileum and colon, respectively; **B and C**, temporal variation in abundances of CAZymes (amylase, cellulase, and hemicellulase), together with genes involved in glycolysis, production of SCFAs (acetate, propionate, and butyrate), and methanogenesis (H_2_ + CO_2_ and methylotrophic pathways); **B** presents the sum of each CAZyme category or metabolism pathway, while **C** presents key rate-limiting enzymes; **D**, graphical overview of early-life assembly trajectory of microbial composition, and carbohydrate metabolic cascades in the rumen and colon during goat development. All the abundances were expressed as transcripts per million (TPM).

The functional potentials of GIT microbiome substantially changed with developmental age (PERMANOVA; *p* < 0.01), with early-stage samples (d1 and d10) forming separate clusters, while later samples (d25, d30 and d90) were more similar, irrespective of GIT region (Fig. 6a). Generally, ruminant GITs possess an efficient microbial polysaccharide degradation system, which comprises of sequential trophic-like metabolic processes of fiber depolymerization, glycolysis, and SCFA production [1]. Herein, we reconstructed carbohydrate metabolism pathways during goat development, using differential microbial KOs and CAZymes. Analysis of CAZymes revealed a gradual increase in the abundances of cellulases (dockerin, GH5, GH9) and hemicellulases (GH43, GH3) as goats aged, irrespective of GIT region (Fig. 6b and 6c). Concurrently, microbial genes involved in glycolysis experienced a slight decrease in the rumen but fluctuated with developmental age in the intestine (Fig. 6b, Additional file 4: Table S17). Notably, acetate production was greater at d1 and d10, when the abundance of the limiting enzyme *acs* in the ileum was >200 times greater (Fig. 6c). Genes involved in butyrate production through acetyl-CoA exhibited a surge in abundance over developmental age in the rumen and colon, but showed the opposite trend in the ileum. In addition, microbial genes involved in propionate biosynthesis via the succinate pathway (*mut*, *epi*, *pct*) were elevated in abundance in the rumen, whereas a greater representation of genes related to propionate biosynthesis via the lactate pathway (*ldh*, *acd*) was observed at d10 and d25. These results suggested that the rumen and colon matured with enhanced microbial carbohydrate-degrading potential (Fig. 6d).

Molecular hydrogen (H_2_) produced during carbohydrate degradation is utilized mainly by methanogenic archaea to facilitate electron transfer through methanogenesis comprising of hydrogenotrophic and methylotrophic pathways [1]. Interestingly, microbial genes involved in these pathways simultaneously exhibited a sharp increase in abundance at d25 after solid feed was offered, and thereafter experienced another surge at d90, irrespective of GIT region (Fig. 6c, Additional file 4: Table S18). Twelve genes encoded by fermentative, electron-bifurcating, energy-converting methanogenic, respiratory, and sensory hydrogenases showed a remarkably consistent increase with methanogenesis (Additional file 2: Fig. S11). Insights into terminal reductases revealed distinct shifts in H_2_ consumption pathways with developmental age, underpinned by increases in methanogenesis, sulfate reduction, and fumarate reduction, but reductions in nitrate ammonification and aerobic respiration (Additional file 4: Table S19). In summary, developmental age and nutrient changes selectively shifted the pathways of H_2_ metabolism, and thereby promoted metabolic cascades to convert polysaccharides into SCFAs in the rumen and colon of goat kids (Fig. 6d).

### Implementation of GKGMC: Modification of metabolic cascades in the ileal microbiome by phytobiotics

**Figure 7:**
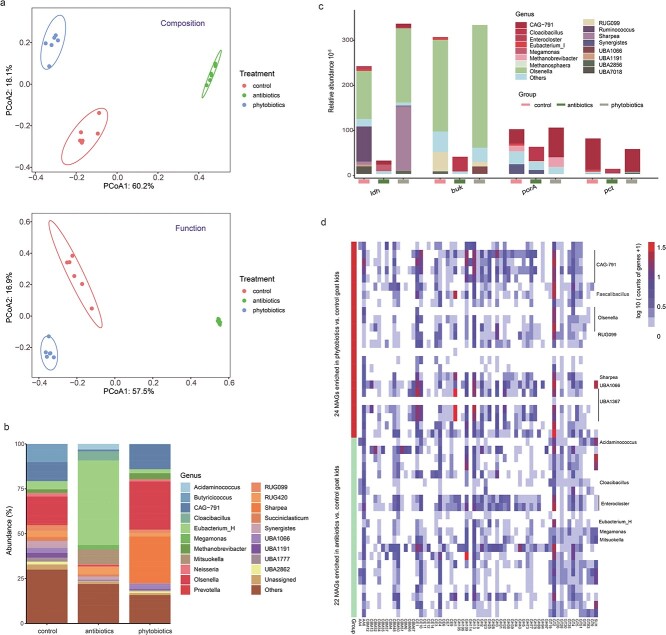
**Modification of metabolic cascades of the ileal microbiome by phytobiotics using the GKGMC as a reference. A**, PCoA analysis of microbial taxonomy and function among control, antibiotics and phytobiotics based on bray–Curtis dissimilarity; **B**, relative abundance of genus-level composition of ileal microbiota; **C**, abundance of genes involved in lactate and butyrate production and their phylogenetic distributions at the genus level; **D**, counts of selected CAZyme-encoding genes in the 46 differentially enriched genomes identified for phytobiotics vs. control, and antibiotics vs. control. The most prevalent assigned genera in these genomes are denoted on the right.

The phytobiotic *M. cordata* extract, with effective ingredients of benzylisoquinoline alkaloids, is a potential alternative to antibiotics for promoting the growth of livestock [55]. Our parallel study asserted that it promoted intestinal immune homeostasis of goat kids, with uppermost changes confined to the small intestine [51]. Herein, with the aid of GKGMC, we aimed to elaborate whether its beneficial effects on hosts were conferred through modulating the gut microbiota. As anticipated, microbial composition and function of these three groups were distinctly separated (Fig. 7a). Members of *Eubacterium_H*, *Mitsuokella*, *Cloacibacillus* and *Megamonas* were significantly enriched within antibiotics group, whereas *Olsenella*, *Sharpea*, and CAG-791 spp*.* were enriched within phytobiotics group (Fig. 7b). Insights from metabolic cascades indicated that the key lactate-metabolism enzyme (*ldh*) was enriched more than 10-fold in phytobiotics group, and was primarily encoded by *Olsenella* and *Sharpea* spp. (Fig. 7c). Likewise, expression of the signature enzyme involved in butyrate biosynthesis (*buk*) exhibited an identical trend, which was predominantly attributed to *Olsenella* spp. Nonetheless, the expression of the genes associated with pyruvate-to-acetyl-CoA interconversion (*porA*) and propionate production (*pct*), decreased sharply, dropping almost 50% in the antibiotics group.

Deeper verification at the MAG level identified 24 and 22 enriched microbial genomes in antibiotics vs. control and phytobiotics vs. control goat kids, respectively (Additional file 5: Tables S20, S21 and S22). Intriguingly, 61-fold enrichment in the genome assigned to *S. azabuensis*, implicated in lactate production and utilization [11], was observed for phytobiotics vs. control goat kids. Concurrently, several MAGs assigned to UBA1367, CAG-791, and *Olsenella* spp. were also selected by phytobiotics vs. control goat kids. CAZyme analysis emphasized the indispensable role of *Sharpea*, UBA1367, and CAG-791 in lactate metabolism, with 5 to 38 lactases of GH1 (Fig. 7d). KO analysis demonstrated that CAG-791 preferred to interconvert pyruvate and acetyl-CoA, and *Olsenella* favored the production of butyrate. To the contrary, antibiotics vs. control goat kids selected MAGs assigned to *Mitsuokella, Megamonas,* and *Eubacterium_H* spp. with more than 15-fold enrichment; these MAGs did not harbor genes for lactate, propionate, or butyrate biosynthesis. Overall, phytobiotics were superior to antibiotics through enrichment of *S. azabuensis* and *Olsenella* spp., which were characterized by lactate formation and utilization in the ileum.

## Discussion

The significance of the GIT microbiome for host physiology and economically important phenotypes highlights the demand for comprehensive analysis based on genomic and phenotypic assays [1]. Herein, we provide a large-scale resource of the GIT microbiome of goat kids, representing 1002 genomes and more than two million proteins, and spanning diverse factors, including developmental age, GIT region, and dietary regime. First, 61.7% of these MAGs appeared to be novel species, suggesting that this GKGMC represents a marginal expansion of available goat microbiome, particularly in goat kids [25, 26, 34]. Second, this catalog substantially expands the diversity and representation of the GIT microbiome by improving the classification rate of metagenomic inventories in goat kids by 25.7% or 51.3% compared with ruminant-associated RGMC or goat-associated GMMC, respectively. Third, GKGMC proteins exhibited low sequence identity with proteins in public KEGG and CAZy databases, indicating the presence of previously uncharacterized function encoded by GIT microorganisms. Overall, this GKGMC serves as an indispensable genomic resource for future studies, specifically for early-life GIT microbiome research in goat kids.

Insights from phylogeny and function of the GKGMC confirm the fact that the goat GIT is home to a diversified anaerobic microbial consortia that synergistically convert plant biomass into value-added products [24]. They harbor a substantial number of CAZymes spanning cellulases, hemicellulases, and pectinases. As anticipated, a high degree of functional redundancy was unveiled for goat kid microbial consortia [24], underpinned by multiple species employing an overlap of carbohydrate-degrading capacity with a similar CAZyme pattern. Notably, our CAZyme inventory also highlights the prospect of fourteen GKGMC-enriched strain-level *Sodaliphilus* spp. as candidates for cellulose degradation with more than sixty cellulosome dockerins. Since there are no cultured representatives of this genus from ruminant origin [6], their reference genomes offer a key step toward cultivation and subsequent discovery of biomass-degrading enzymes for biotechnological applications.

Fecal microbiome has long been regarded as a proxy for the GIT microbiota owing to its noninvasive collection nature in humans [56]. However, using the GKGMC, we uncovered pronounced spatial heterogeneity as a hallmark of GIT microorganisms independent of developmental age in goat kids, which coincides with the previous notion of GIT biogeography of microbiota in humans [57], monogastric animals [50], and ruminants [12, 58]. All these findings suggest that the entire GIT should be taken in account in subsequent microbial ecology research [12]. From an ecological perspective, the spatially stratified niches of the GIT varying in physicochemical and nutrient gradients, as well as compartmentalized host immune activity, facilitate greater taxonomic and metabolic diversity of resident microbial consortia [57]. Ruminal microorganisms specialized in efficient degradation of complex plant polysaccharides, underpinned by the prevalence of fiber- and starch-degrading members, as well as the enrichment of genes involved in plant depolymerization and SCFA production [1]. Moreover, the colon preferentially selected *Bacteroides* spp. and exhibited similar abundances of CAZymes as did the rumen. This finding suggests that microbial fermentation in the hindgut complements fermentation in the foregut, where the microbes encounter recalcitrant polysaccharides that are not completely processed [24]. In contrast, the rapid uptake and conversion of available simple sugars contribute to maintaining the microbial ecosystem in the ileum, and their metabolic apparatus facilitates survival in harsh environments with low pH, rapid luminal flow, and secretion of bactericidal bile acids [57] .

Given the materiality of early-life GIT microbiota for long-term health and productivity of animals [18, 59], we further elaborated their developmental trajectories as goats matured. As anticipated, microbial consortia colonized rapidly and dynamically in the GIT of goats after birth. From an ecological perspective, the early-stage microbiota should be more receptive to interventions than adult microbiota owing to its lower diversity and colonization resistance [20]. In addition, late-successional microbes attempting to colonize strongly depend upon niches established by early-arriving species, known as historical contingency [60]. Analysis of microbial assembly dynamics claimed that biotic factors (historical contingency) and abiotic factors (including developmental age, GIT region, and dietary regime) jointly contributed to the deterministic homogeneous selection process (Additional file 1) [59]. Furthermore, their compositional and functional diversity exhibited three phases of progression, following consistent trajectories with dynamic changes in developmental age (nonrumination, transition, and rumination) and dietary regime (breastfeeding to solid feed), similar to previous observations in humans [18, 60] and cows [9, 59]. This pattern consistency across different animal-associated communities may be indicative of a more fundamental developmental phase during host development, and might provide clues for human growth. Ultimately, we comprehensively depicted the carbohydrate metabolic landscapes by revealing which, where, when, and how GIT microorganisms coevolved with the capacity to harvest energy from recalcitrant dietary polysaccharides, and shift H_2_ metabolism through methanogenesis. The use of these enzymatic apparatuses and metabolic pathways in the GIT microbial consortia of goat kids might create opportunities to develop sustainable carbohydrate-based technologies [1].

Mining for novel ideal substitutes for antibiotics in animal production is vital during the new global era of nonantibiotic growth promoters, with great efforts have been made on phytobiotics [55]. With aid of the GKGMC, we uncovered their health-promoting effect was achieved by promoting the beneficial lactate-producer *S. azabuensis* and lactate-utilizer *Olsenella* spp in the ileum of goat kids, which competitively inhibited pathogen colonization. Similarly, phytobiotics enhanced the predominant role of the lactate-producer *Lactobacillus*, and upregulated the biosynthesis pathways of amino acids, vitamins, and secondary bile acids in the foregut of chicken [55]. These findings underscore the importance of microbial lactate metabolism in response to phytobiotics, and suggest that host genetics should be considered when promoting health through phytobiotics.

## Conclusions

We presented the goat kid-specific GKGMC dataset covering three compartments spatially and five developmental ages temporally, and remarkably extended the GIT microbial taxonomic and functional characteristics of goat kids. Using the GKGMC, we first deciphered the biogeography of the GIT microbiota. Second, GIT microorganisms were rapidly assembled after birth, and developmental age and nutrient changes selectively drove microbial H_2_ metabolism and thereby promoted metabolic cascades to convert polysaccharides into SCFAs in the rumen and colon. Third, this GKGMC proved to be useful for revealing the microbial mechanism through which phytobiotics promoted intestinal immune homeostasis in goat kids. This GKGMC provides an invaluable stepping stone to the cultivation of previously unculturable microbial taxa, and is of utmost materiality for future studies to improve productivity and health through the precise manipulation of the GIT microorganisms in goat kids.

## Supplementary Material

Additional_file_1_Supplemental_Text_wrae002

Additional_file_2_Supplemetntal_figures_1_11_wrae002

Additional_file_3_Supplemental_Tables_1_12_wrae002

Additional_file_4_Supplemental_Tables_13_19_wrae002

Additional_file_5_Supplemental_Tables_20_22_wrae002

Description_of_additional_supplementary_files_wrae002

## Data Availability

Metagenomic sequencing data have been deposited in the China National GeneBank Database with accession number CNP0004363. The GKGMC are publicly available in the Figshare database (https://doi.org/10.6084/m9.figshare.24559456).
