# Supplementary material for: Ecological niches and assembly dynamics of diverse microbial consortia in the gastrointestine of goat kids
Source: ISME J. 2024 Jan 11;18(1):wrae002. doi: 10.1093/ismejo/wrae002 (PMC10872696; doi:10.1093/ismejo/wrae002)
Supplement: Additional_file_1_Supplemental_Text_wrae002 [file additional_file_1_supplemental_text_wrae002.pdf]

Supplemental Text

MATERIALS AND METHODS

Profiling of SCFAs in the GIT contents

Three grams of GIT contents were collected for profiling of SCFAs according to the procedure detailed in our previous study [1]. The SCFA concentrations (acetate, propionate and butyrate) of the GIT contents were assayed using a gas chromatographer (Agilent 7890A, Agilent Inc., Palo Alto, CA) following the procedure as previously described by Jiao et al., (2015) [1]. The molar proportion of individual SCFA to the total SCFA concentration was calculated.

Histomorphological assessment of GIT tissues

After the GIT was emptied of its contents, it was rinsed with prechilled phosphate buffer solution (pH = 7.4) for several times, and tissue samples were collected with a shape scalpel, and then immersed in a 10% formalin solution immediately for further analysis [1]. The histomorphological structure of the rumen, ileum and colon tissues were assessed using the hematoxylin and eosin staining method as detailed in our previous study [1]. The rumen indicators included rumen papillae height, width and area. The ileum indicators included villus height, crypt depth, whereas the colonic mucosal thickness and muscle layer was measured.

Microbial co-occurrence network analysis

To estimate microbial coexistence, the Spearman's correlations between each MAG pair were calculated based on their abundances across our sampling cohort

using R package Hmisc. Robust correlations with correlation coefficients (ρ) > 0.80 and false discovery rate (*fdr*) < 0.01 were selected to construct networks. The node and edge files were imported into Gephi [2] to graph networks. The topological features, including degree, betweenness centrality, closeness centrality and eigencentrality, were calculated for each node. The hub MAGs were defined as those nodes with the highest eigencentrality connectivity in the network.

Phylogenetic analysis of *Treponema*, F082, *Ruminococcus_E*, *Sodaliphilus*, and *Archaea*

PhyloPhlAn (v.1.0) was used to build the phylogenetic tree of these genera of concern [3]. The reference genome sequences were from NCBI with GenBank ID listed in Additional file 3: Table S9. Afterwards, the phylogenetic tree was visualized with aid of Interactive Tree Of Life (iTOL) [4].

Analysis of microbial community assembly processes

We further deciphered the microbial community assembly mechanisms with aid of phylogenetic bin based null model analysis (iCAMP) [5]. Phylogenetic similarity was calculated as beta net relatedness index (βNRI), and compositional turnover was quantified by the Raup-Crick index (RC). The fraction of pairwise comparisons with βNRI < −1.96 is considered as the percentages of homogeneous selection (HoS), whereas those with βNRI > +1.96 as the percentages of heterogeneous selection (HeS). Moreover, the fraction of pairwise comparisons with |βNRI| ≤ 1.96, RC < −0.95 is treated as the percentages of homogenizing dispersal (HD), those with |βNRI|

≤ 1.96, RC > + 0.95 as dispersal limitation (DL), whereas those with |βNRI| ≤ 1.96 and |RC| ≤ 0.95 as drift (DR) [5].

Statistical analysis

The data of growth performance, GIT morphological parameters and SCFAs at different developmental ages were analyzed with one way ANOVA and Duncan's multiple comparisons.

RESULTS

Gastrointestinal morphological maturation and microbial functional establishment during animal development in goat kids

The body weight (BW) and carcass weight (CW) of goats increased ($p < 0.01$) substantially over time, with a slight drop after weaning at d30, reaching 8.5 ± 1.1 and 3.1 ± 0.5 kg at d90, respectively (Additional file 2: Fig. S1a). Similarly, the oblique body length, body height and chest girth experienced a synchronous increase ($p < 0.01$) over time and reached 45.4, 41.4 and 48.1 cm at d90, respectively.

The goat GIT exhibits morphological, functional and microbial changes after birth, which jointly contribute to its maturation, and ultimately animal performance and health status [1]. As anticipated, insights from rumen papillae indicated a progressive increment in its length, width and area during animal development ($p < 0.05$, Additional file 2: Fig. S1b). Of note, the rumen papillae enlarges most rapidly after weaning when the liquid diet is replaced by the solid diet, with its length, width

and area at d90 was 3.2, 1.4 and 4.7 times as those at d30. In term of small intestine, the morphology of intestinal villus and crypt is an important indicator for evaluating intestinal health status and nutrient absorptive capacity. The ileal villus height and crypt depth did not show significant differences from birth to d30, whereas skyrocketed by 42% and 85% afterwards to d90, respectively ($p < 0.05$, Additional file 2: Fig. S1c). In regard to the colon, the mucosal thickness and muscle layer thickness experienced a remarkable increase at d25, stabilized at d30, and skyrocketed by 36% and 18% at d90 ($p < 0.05$, Additional file 2: Fig. S1d).

Short-chain fatty acids (SCFAs), produced during microbial fermentation of dietary carbohydrates in the GIT, are promising key metabolites mediating microbiota-host relationships. The SCFA concentration in the GIT digesta of goats were not detectable at birth, hence the initial SCFA pattern, dynamics and equilibria were characterised from d10 to d90. Acetate was the only detectable SCFA in the ileum, and its concentration remained stable during d25 to d90 (Additional file 2: Fig. S1e, $p > 0.05$). As age increased, the concentration of total SCFA in the rumen and colon gradually raised from 22.8 mM and 28.8 mM at d10, up to 59.5 mM and 44.7 mM at d90, respectively ($p < 0.01$). With respect to SCFA profile, it showed similar shifts from acetate production towards propionate and butyrate production in both rumen (Additional file 2: Fig. S1f) and colon (Additional file 2: Fig. S1g) from d10 to d90, as acetate molar proportion decreased remarkably ($p < 0.01$), while molar

proportions of propionate and butyrate increased by 63.9% and 163.5% in the rumen, by 78.5% and 25.0% in the colon, respectively ($p < 0.01$).

Co-occurrence network of 1,002 MAGs

Knowledge from co-occurrence network the indicated that these MAGs were primarily divided into three distinct regional groups, confirming the specialized distributive heterogeneity of the GIT microbiota (Additional file 2: Fig. S9). Further network topological features indicated that the rumen exhibited the highest degree and betweenness centrality scores, followed by the colon, significantly higher than those of the ileum, suggesting microbial species co-occurred and interconnected more frequently within the rumen and colon. Insights from the hub microbes suggested that *Treponema* and CAG-74 in the rumen, UBA1066 and *Eubacterium_A* in the ileum, and *Faecalibacterium* and UBA2882 in the colon served as hub populations responsible for maintaining the balance and function of networks.

Both deterministic and stochastic processes orchestrate compositional plasticity and species coexistence of GIT microorganisms

Revealing the processes that drive microbial community assembly is a fundamental but scarcely investigated topic in microbial ecology of goats. It is of particular significance to quantify the relative importance of stochastic (birth, death, colonization, extinction and speciation) and deterministic (species biological interactions and environmental conditions) processes in governing community diversity, structure, and succession [6]. Based on iCAMP, stochasticity is comprised

of homogenizing dispersal (HD), dispersal limitation (DL) and drift (DR), while determinism includes homogeneous selection (HoS) and heterogeneous selection (HeS) [5]. In in all GIT regions, DL and HoS were more important than other ecological processes in shaping microbial community assembly, with average relative importance of 54.8%-63.4% and 24.1%-36.8%, respectively (Additional file 2: Fig. S10). The HoS dominated (>70%) microbial assembly at d0, experienced a sharp decrease at d10 in the rumen and colon whereas a slight decrease at d10 in the ileum, thereafter remaining stable after solid feed offered. The relative importance of DL showed a U-shaped pattern as goats aged. Despite HeS was always of minor importance in the colon at all ages, it accounted for 12.1% and 10.0% of relative importance in the rumen and ileum at d30. In summary, microbial assembly dynamics were orchestrated by fine-scale deterministic and stochastic ecological processes in GIT of goat kids.

DISCUSSION

Understanding the ecological mechanisms driving microbial assembly dynamics is critical for the mechanistic comprehension of biodiversity maintenance, community stability, and ecosystem functioning [6]. Our findings underscore the fine-scale joint contribution of stochastic and deterministic processes in driving the assembly of microbial consortia in the GIT of goat kids, with priority manifesting the dominant roles of dispersal limitation and homogeneous selection. In community ecology

research, homogeneous selection refers to selection under homogeneous abiotic and biotic environmental conditions, leading to more similar structures among communities. Whereas, dispersal limitation is considered to be when the migration of an organism to a new location is limited due to certain restrictions, leading to more dissimilar structures among communities [5, 6]. Herein, the deterministic homogeneous selection could be imposed by abiotic factors (including developmental age, gut region, dietary regime) and biotic factors (historical contingency, of which pioneer microbes might exert a hindering or facilitating impact on the late-arriving species) [6, 7]. Similar assembly dynamics has been reported to the rumen of cows, with the exception that historical contingency was regarded as stochasticity [7]. The GIT microbial assembly pattern at d1 was dominantly governed by homogeneous selection, suggesting that the seed of microbial consortia is substantially shaped by the deterministic factors, with suspected emphasis on mother-infant transmission and environment exposure [8]. Afterwards, the homogeneous selection was stepwise replaced by the dispersal limitation from d10 to d90. Likewise, the relative importance of stochastic processes decreased over succession of microbial communities in the zebrafish [9]. Ultimately, this pattern consistency across different animal-associated communities may be indicative of more fundamental ecological processes over host development, and might provide clues for human growth. This study is based on phylogenetic diversity, and deeper functional-trait-based microbial assembly researches are warranted with urgent need of new statistical approaches [6].

REFERENCES

1. Jiao J, Li X, Beauchemin KA, Tan Z, Tang S, Zhou C: Rumen development process in goats as affected by supplemental feeding v. grazing: age-related anatomic development, functional achievement and microbial colonisation. Br J Nutr 2015; 113:888-900.
2. Bastian M., Heymann S., M J: Gephi : An open source software for exploring and manipulating networks. International AAAI Conference on Weblogs and Social Media 2009.
3. Asnicar F, Thomas AM, Beghini F, Mengoni C, Manara S, Manghi P, et al: Precise phylogenetic analysis of microbial isolates and genomes from metagenomes using PhyloPhlAn 3.0. Nat Commun 2020; 11.
4. Letunic I, Bork P: Interactive Tree Of Life (iTOL) v5: an online tool for phylogenetic tree display and annotation. Nucleic Acids Res 2021; 49:W293-W296.
5. Ning D, Yuan M, Wu L, Zhang Y, Guo X, Zhou X, et al: A quantitative framework reveals ecological drivers of grassland microbial community assembly in response to warming. Nat Commun 2020; 11:4717.
6. Zhou J, Ning D: Stochastic community assembly: does it matter in microbial ecology? Microbiol Mol Biol Rev 2017; 81.
7. Furman O, Shenhav L, Sasson G, Kokou F, Honig H, Jacoby S, et al: Stochasticity constrained by deterministic effects of diet and age drive rumen microbiome assembly dynamics. Nat Commun 2020; 11:1904.
8. Browne HP, Shao Y, Lawley TD: Mother-infant transmission of human microbiota. Curr Opin Microbiol 2022; 69:102173.
9. Burns AR, Stephens WZ, Stagaman K, Wong S, Rawls JF, Guillemin K, et al: Contribution of neutral processes to the assembly of gut microbial communities in the zebrafish over host development. ISME J 2016; 10:655-664.
