# Supplementary material for: Ecological niches and assembly dynamics of diverse microbial consortia in the gastrointestine of goat kids
Source: ISME J. 2024 Jan 11;18(1):wrae002. doi: 10.1093/ismejo/wrae002 (PMC10872696; doi:10.1093/ismejo/wrae002)
Supplement: Additional_file_2_Supplemetntal_figures_1_11_wrae002 [file additional_file_2_supplemetntal_figures_1_11_wrae002.pdf]

Supplemental figure legends

Fig. S1. Growth performance, gastrointestinal morphology and SCFAs of goat kids at different developmental ages.

a, Growth parameters of goats, including body height, body length, chest grith, live weight and carcass weight; **b,** Inspection of morphological changes in rumen papillae; **c,** Inspection of morphological changes in ileum villus and crypt; **d,** Inspection of morphological changes in colon mucosa and muscle; **e,** Total short chain fatty acids in the rumen, ileum and colon; **f** and **f**, Short chain fatty profile in the rumen and colon.

Fig. S2. Generation of the ruminant GIT microbial catalog (RGMC) from seven public available large cohort studies in ruminants and Hungate collection genomes.

Fig. S3. Percentage increase of the proportion of reads, partitioned by GIT region or developmental age that were assigned to the GKGMC (goat kid GIT microbial catalog) , GKGMC plus RGMC, in relation to RGMC (**a**), that were assigned to the GKGMC, GKGMC plus GMMC (Goat multi-kingdom microbial catalog), in relation to GMMC (**b**).

Fig. S4. Distribution of microorganisms at phylum-level among four microbial genome catalogs (RGMC, GMMC, GFMC and GKGMC in this study).

GFMC, goat fecal microbial catalog.

Fig. S5. Distribution of microorganisms at genus-level among four microbial genome catalogs (RGMC, GMMC, GFMC and GKGMC in this study).

Fig. S6. Functional annotation of GKGMC against KEGG, eggNOG and CAZy databases.

Fig. S7. Distribution of different classes of metabolic gene clusters (MGCs) across

dominant phyla in GKGMC based on gutSMASH.

Fig. S8. PCoA analysis of GIT effect on microbial composition and function based on bray-curtis dissimilarity.

Fig. S9. Co-occurrence interaction network of the 1,002 MAGs based on Spearman correlation indices calculated from the abundances of MAGs in each sample, in three GIT regions (**a**) or individually (**b**).
The colors of the nodes indicate GIT regions (**a**) and network modules (**b**).

Fig. S10. Analysis of microbial assembly dynamics using iCAMP.
a. The relative importance of different ecological processes in the rumen, ileum and colon, respectively. **b.** Changes in the relative importance of five different ecological processes in the GIT as goat matured.

Fig. S11. Changes in microbial carbohydrate-related functional maturation in the GIT microbiota of goat kids from birth to rumination.
a, Temporal variation in abundance of CAZymes involved in plant depolymerization; Temporal variation in abundance of genes involved in glycolysis **(b)**, SCFA production **(c)** and methanogenesis **(d)**; Temporal variation in abundance of hydrogenases **(e)** and related terminal reductases **(f).**
All the abundances were expressed as transcripts per million (TPM).

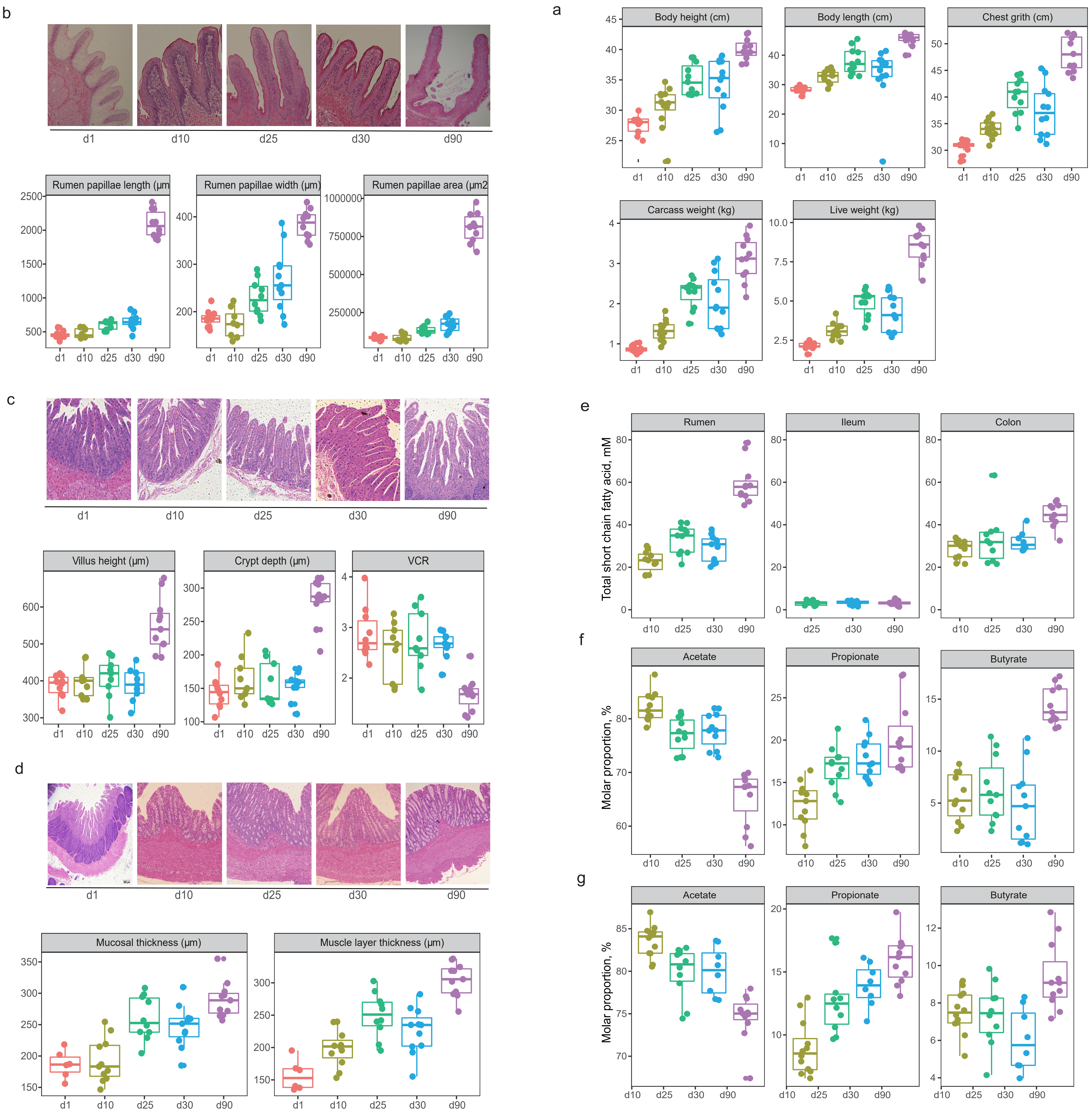

b
d1
d10
d25
d30
d90
Rumen papillae length (μm
Rumen papillae width (μm)
Rumen papillae area (μm2
a
Body height (cm)
Body length (cm)
Chest grith (cm)
Carcass weight (kg)
Live weight (kg)
c
Villus height (μm)
Crypt depth (μm)
VCR
d
Mucosal thickness (μm)
Muscle layer thickness (μm)
e
Total short chain fatty acid, mM
Rumen
Ileum
Colon
f
Molar proportion, %
Acetate
Propionate
Butyrate
g
Molar proportion, %
Acetate
Propionate
Butyrate

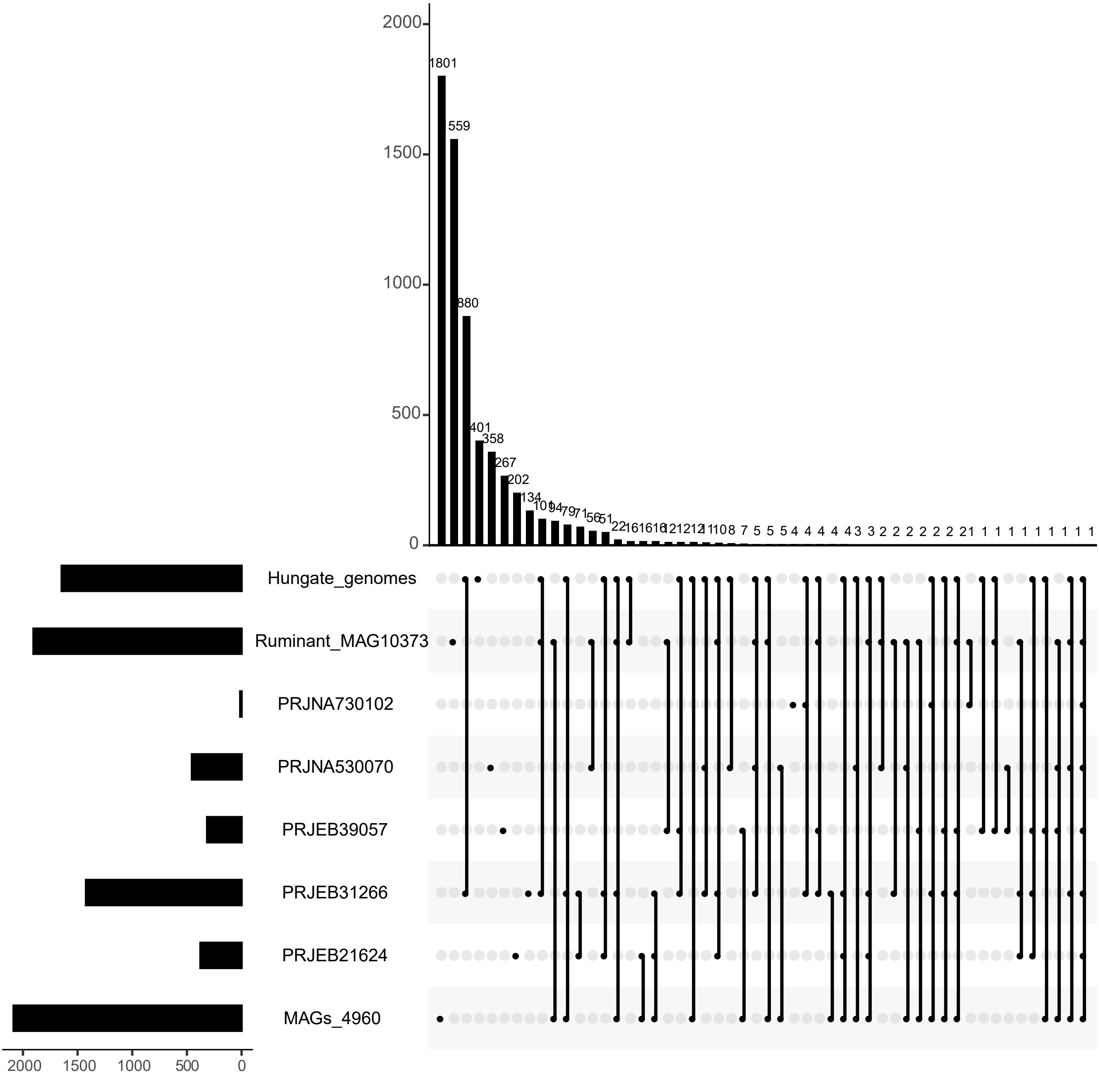

2000
1500
1000
500
0
1801
559
880
401
358
267
202
134
101
94
79
71
56
51
22
Hungate_genomes
Ruminant_MAG10373
PRJNA730102
PRJNA530070
PRJEB39057
PRJEB31266
PRJEB21624
MAGs_4960
2000
1500
1000
500
0

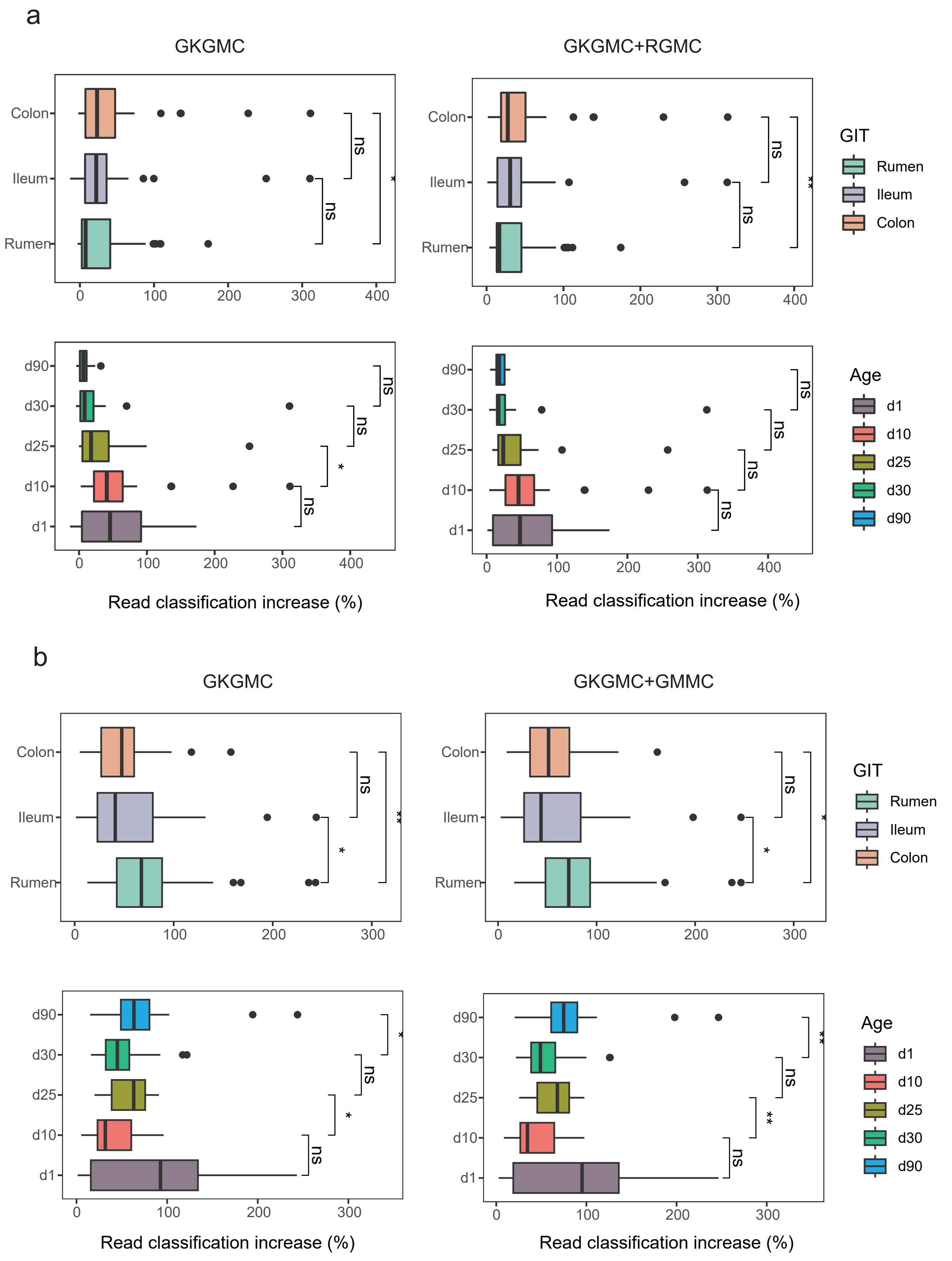

a
GKGMC
GKGMC+RGMC
Colon
Ileum
Rumen
ns
ns
*
**
0
100
200
300
400
GIT
Rumen
Ileum
Colon
d90
d30
d25
d10
d1
Age
d1
d10
d25
d30
d90
Read classification increase (%)
b
GKGMC
GKGMC+GMMC
Read classification increase (%)

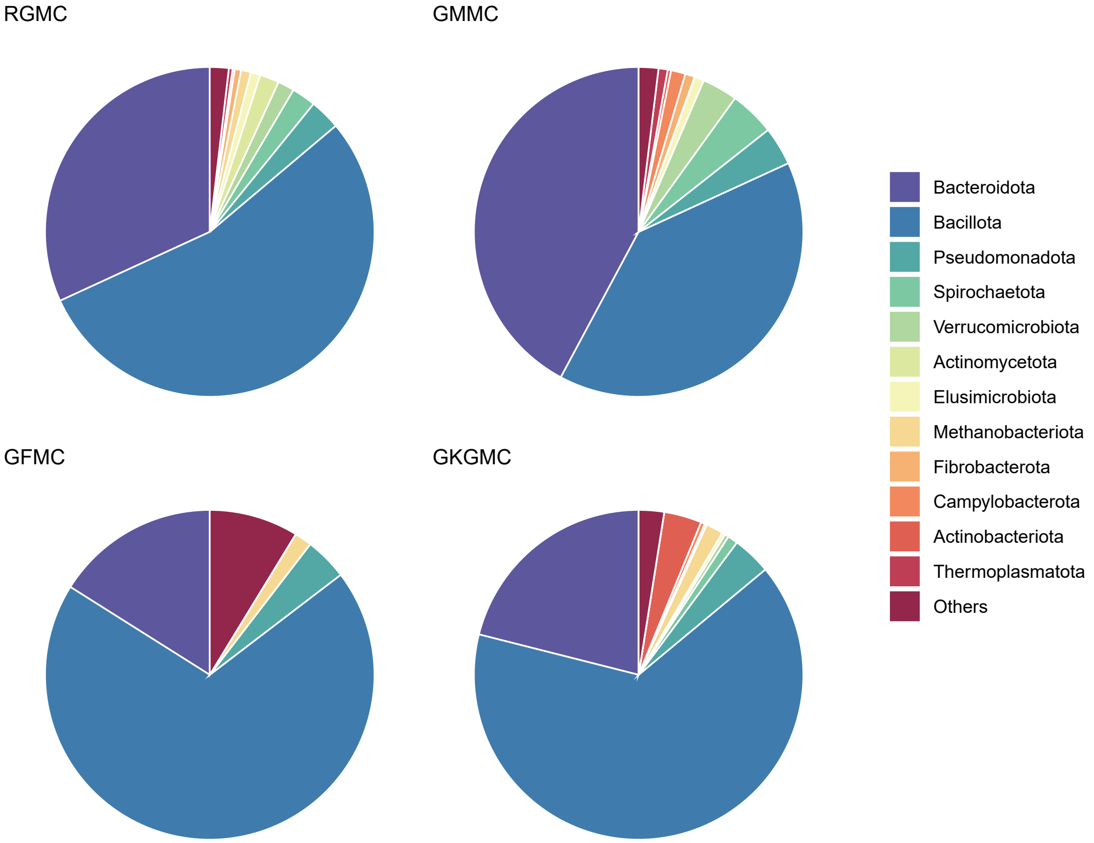
RGMC
GMMC
GFMC
GKGMC
Bacteroidota
Bacillota
Pseudomonadota
Spirochaetota
Verrucomicrobiota
Actinomycetota
Elusimicrobiota
Methanobacteriota
Fibrobacterota
Campylobacterota
Actinobacteriota
Thermoplasmatota
Others

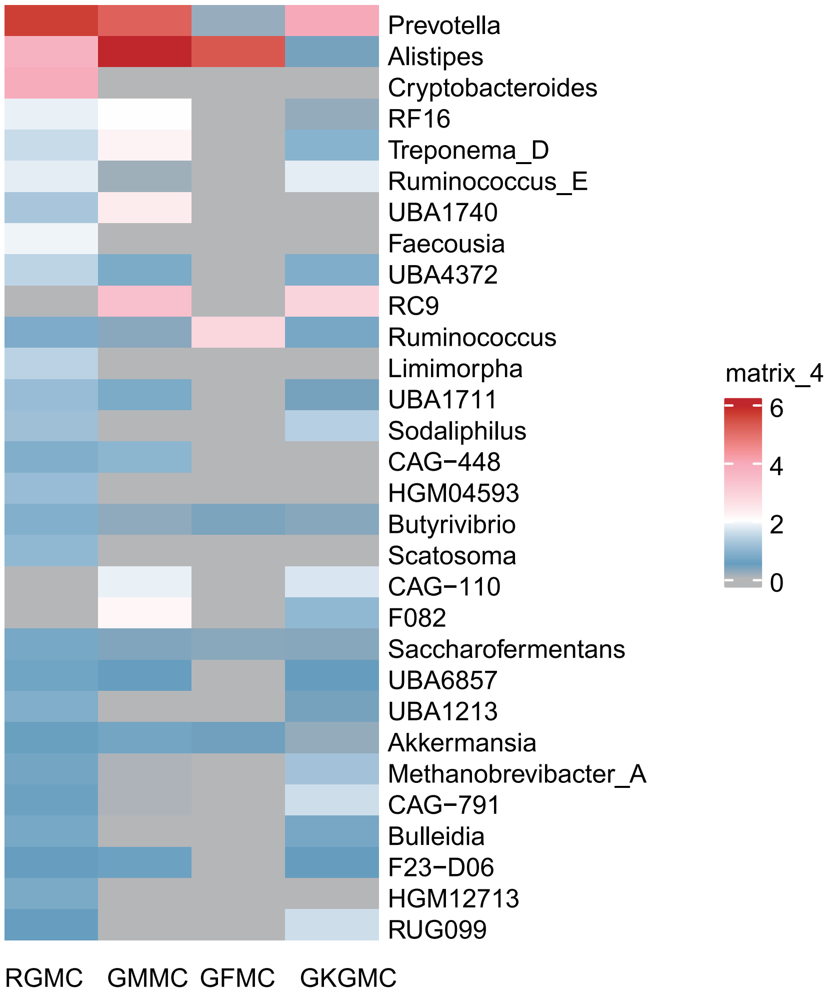
Prevotella
Alistipes
Cryptobacteroides
RF16
Treponema_D
Ruminococcus_E
UBA1740
Faecousia
UBA4372
RC9
Ruminococcus
Limimorpha
UBA1711
Sodaliphilus
CAG−448
HGM04593
Butyrivibrio
Scatosoma
CAG−110
F082
Saccharofermentans
UBA6857
UBA1213
Akkermansia
Methanobrevibacter_A
CAG−791
Bulleidia
F23−D06
HGM12713
RUG099
RGMC
GMMC
GFMC
GKGMC
matrix_4
6
4
2
0

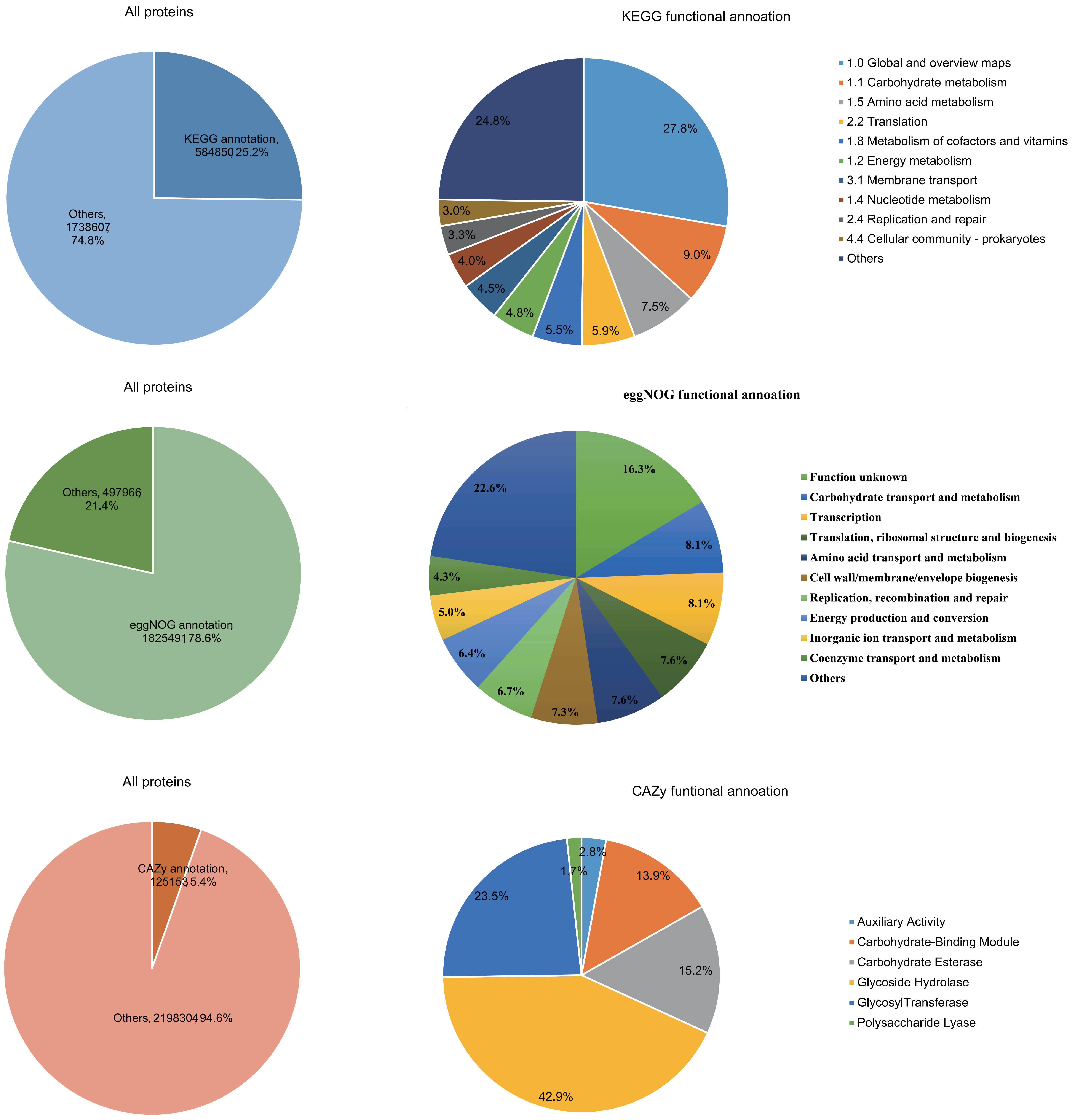
All proteins
KEGG annotation, 584850, 25.2%
Others, 1738607, 74.8%
KEGG functional annoation
27.8%
9.0%
7.5%
5.9%
5.5%
4.8%
4.5%
4.0%
3.3%
3.0%
24.8%
1.0 Global and overview maps
1.1 Carbohydrate metabolism
1.5 Amino acid metabolism
2.2 Translation
1.8 Metabolism of cofactors and vitamins
1.2 Energy metabolism
3.1 Membrane transport
1.4 Nucleotide metabolism
2.4 Replication and repair
4.4 Cellular community - prokaryotes
Others
All proteins
Others, 497966, 21.4%
eggNOG annotation, 1825491, 78.6%
eggNOG functional annoation
16.3%
8.1%
8.1%
7.6%
7.6%
7.3%
6.7%
6.4%
5.0%
4.3%
22.6%
Function unknown
Carbohydrate transport and metabolism
Transcription
Translation, ribosomal structure and biogenesis
Amino acid transport and metabolism
Cell wall/membrane/envelope biogenesis
Replication, recombination and repair
Energy production and conversion
Inorganic ion transport and metabolism
Coenzyme transport and metabolism
Others
All proteins
CAZy annotation, 125153, 5.4%
Others, 2198304, 94.6%
CAZy funtional annoation
2.8%
13.9%
15.2%
42.9%
23.5%
1.7%
Auxiliary Activity
Carbohydrate-Binding Module
Carbohydrate Esterase
Glycoside Hydrolase
GlycosylTransferase
Polysaccharide Lyase

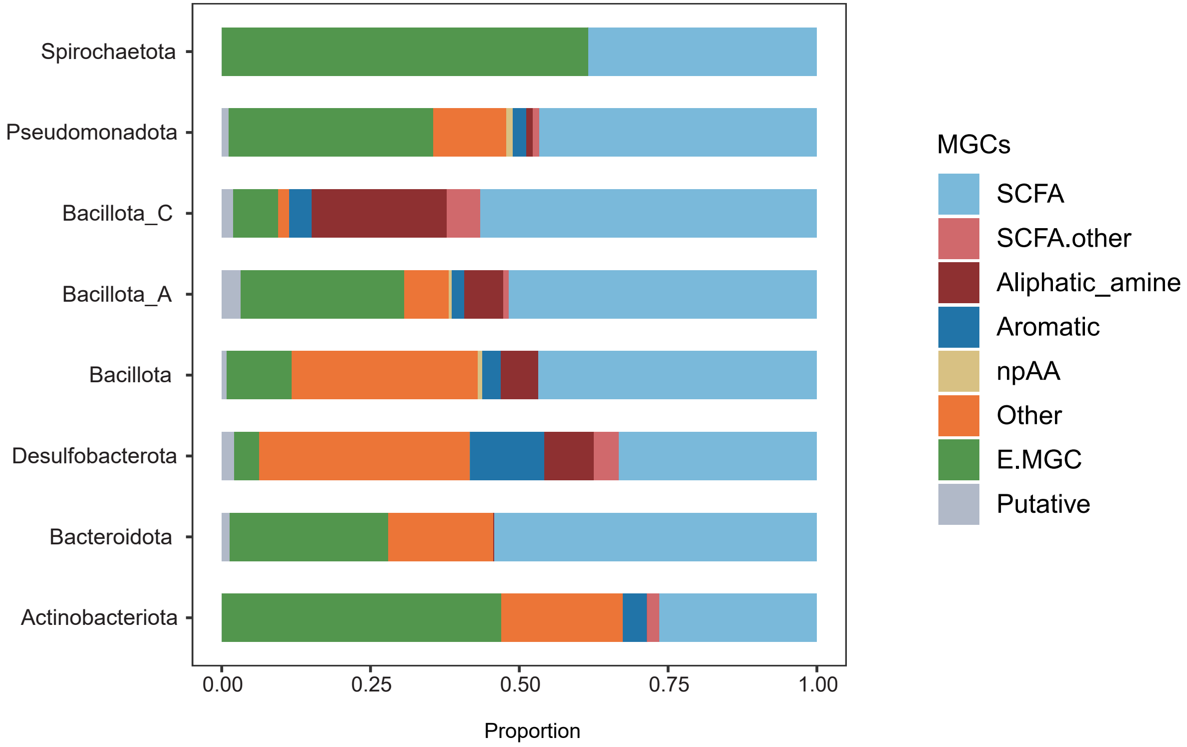
Spirochaetota
Pseudomonadota
Bacillota_C
Bacillota_A
Bacillota
Desulfobacterota
Bacteroidota
Actinobacteriota
0.00
0.25
0.50
0.75
1.00
Proportion
MGCs
SCFA
SCFA.other
Aliphatic_amine
Aromatic
npAA
Other
E.MGC
Putative

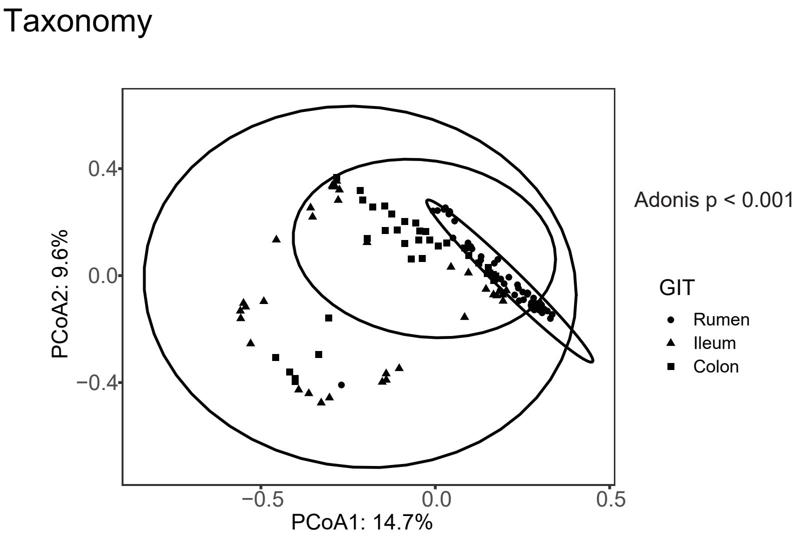
Taxonomy
0.4
0.0
−0.4
PCoA2: 9.6%
−0.5
0.0
0.5
PCoA1: 14.7%
Adonis p < 0.001
GIT
Rumen
Ileum
Colon

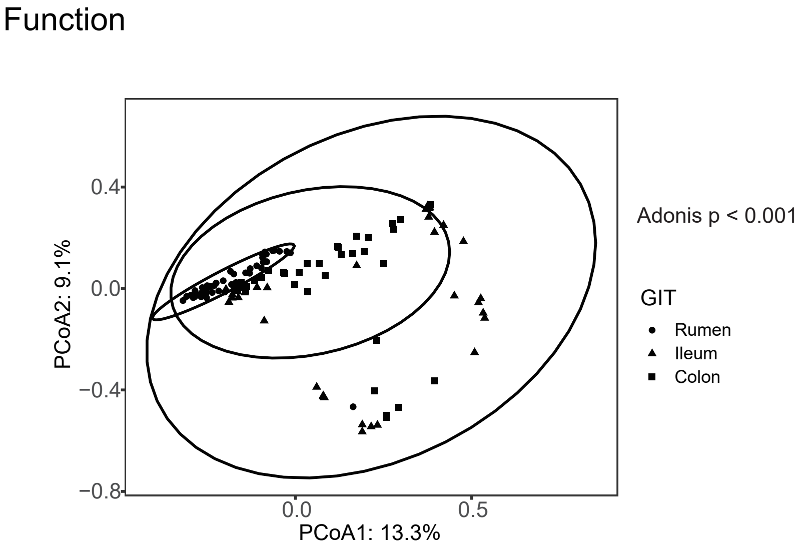
Function
0.4
0.0
−0.4
−0.8
PCoA2: 9.1%
0.0
0.5
PCoA1: 13.3%
Adonis p < 0.001
GIT
Rumen
Ileum
Colon

a

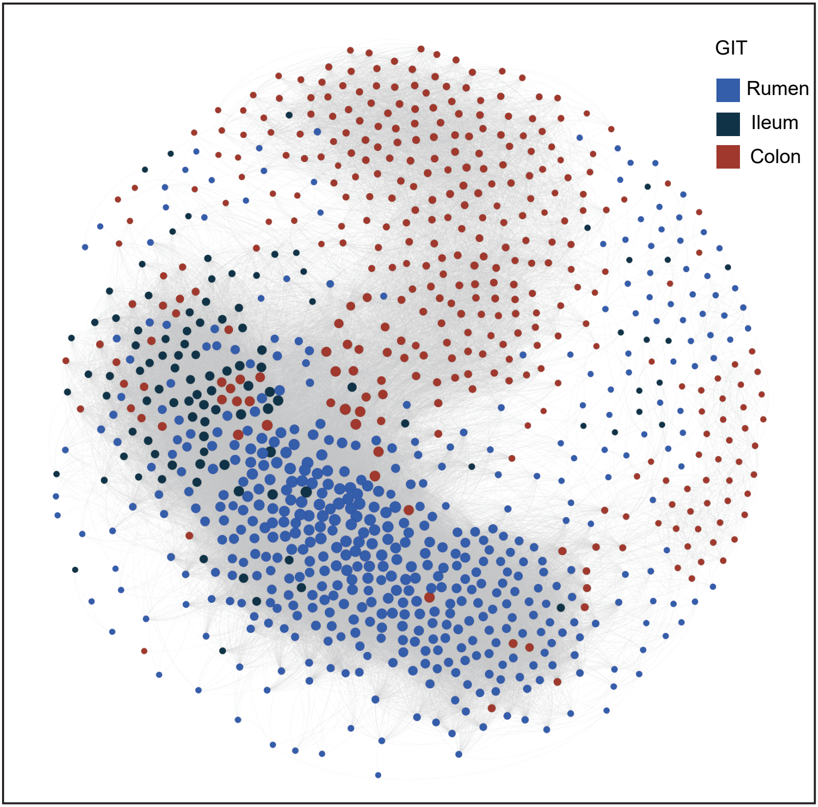

b

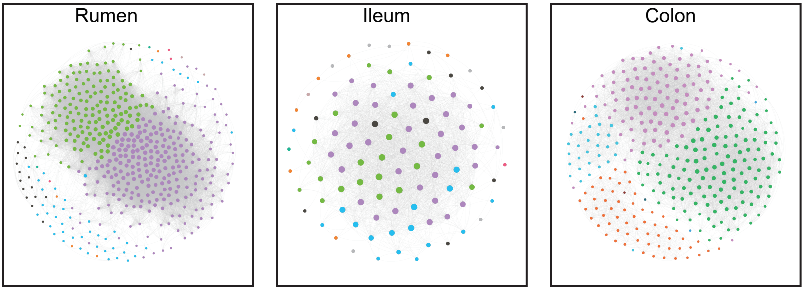

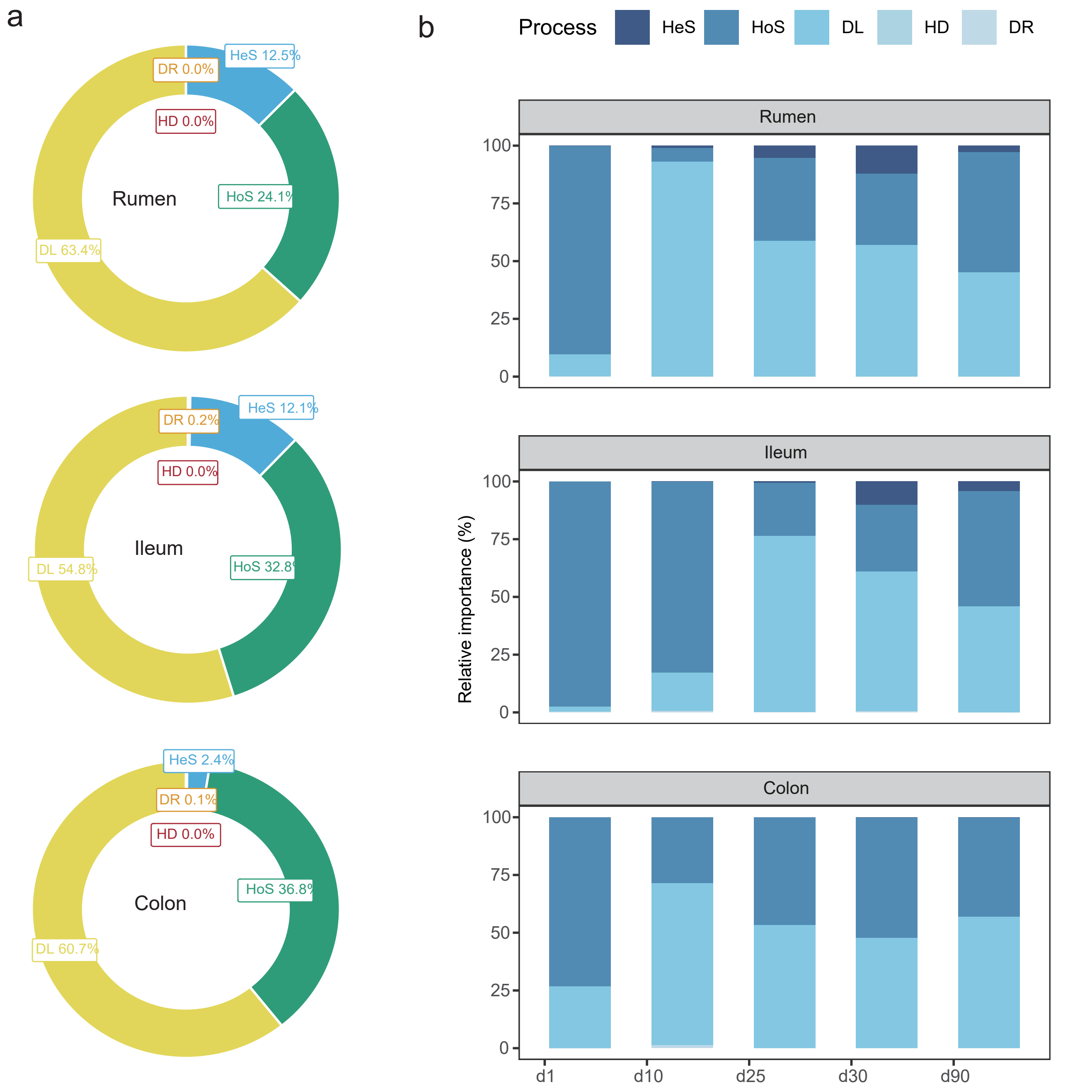
a
Rumen
HeS 12.5%
DR 0.0%
HD 0.0%
HoS 24.1%
DL 63.4%
Ileum
HeS 12.1%
DR 0.2%
HD 0.0%
HoS 32.8%
DL 54.8%
Colon
HeS 2.4%
DR 0.1%
HD 0.0%
HoS 36.8%
DL 60.7%
b
Process
HeS
HoS
DL
HD
DR
Rumen
Ileum
Colon
Relative importance (%)
0
25
50
75
100
d1
d10
d25
d30
d90

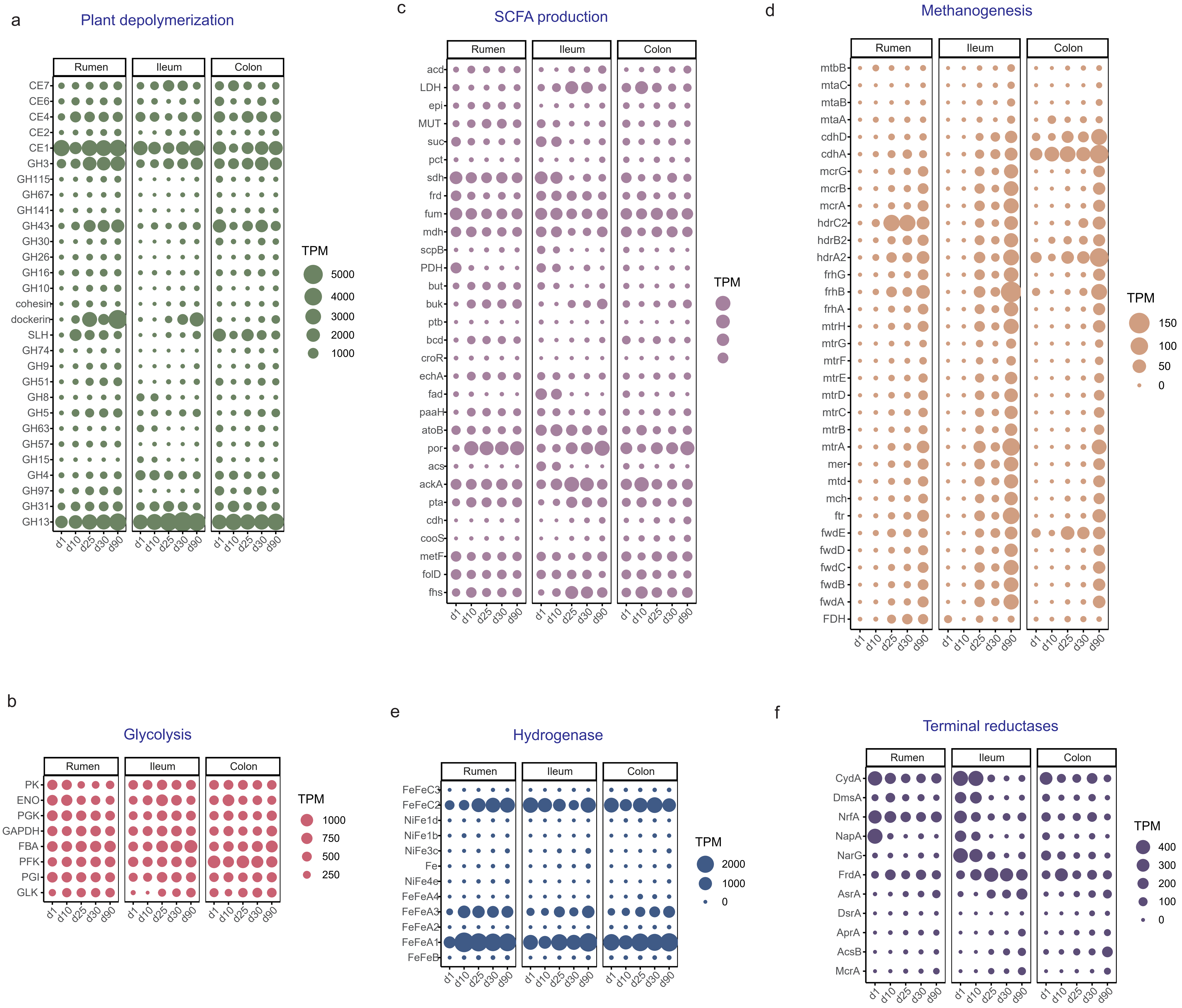
a
Plant depolymerization
Rumen
Ileum
Colon
CE7
CE6
CE4
CE2
CE1
GH3
GH115
GH67
GH141
GH43
GH30
GH26
GH16
GH10
cohesin
dockerin
SLH
GH74
GH9
GH51
GH8
GH5
GH63
GH57
GH15
GH4
GH97
GH31
GH13
d1
d10
d25
d30
d90
TPM
5000
4000
3000
2000
1000
c
SCFA production
acd
LDH
epi
MUT
suc
pct
sdh
frd
fum
mdh
scpB
PDH
but
buk
ptb
bcd
croR
echA
fad
paaH
atoB
por
acs
ackA
pta
cdh
cooS
metF
folD
fhs
TPM
d
Methanogenesis
mtbB
mtaC
mtaB
mtaA
cdhD
cdhA
mcrG
mcrB
mcrA
hdrC2
hdrB2
hdrA2
frhG
frhB
frhA
mtrH
mtrG
mtrF
mtrE
mtrD
mtrC
mtrB
mtrA
mer
mtd
mch
ftr
fwdE
fwdD
fwdC
fwdB
fwdA
FDH
TPM
150
100
50
0
b
Glycolysis
PK
ENO
PGK
GAPDH
FBA
PFK
PGI
GLK
TPM
1000
750
500
250
e
Hydrogenase
FeFeC3
FeFeC2
NiFe1d
NiFe1b
NiFe3c
Fe
NiFe4e
FeFeA4
FeFeA3
FeFeA2
FeFeA1
FeFeB
TPM
2000
1000
0
f
Terminal reductases
CydA
DmsA
NrfA
NapA
NarG
FrdA
AsrA
DsrA
AprA
AcsB
McrA
TPM
400
300
200
100
0
