# Supplementary material for: Ecological niches and assembly dynamics of diverse microbial consortia in the gastrointestine of goat kids
Source: ISME J. 2024 Jan 11;18(1):wrae002. doi: 10.1093/ismejo/wrae002 (PMC10872696; doi:10.1093/ismejo/wrae002)
Supplement: Description_of_additional_supplementary_files_wrae002 [file description_of_additional_supplementary_files_wrae002.pdf]

Description of additional supplementary files

Additional file 1. Supplemental Text.

Additional file 2. Supplemental Figures 1 to 11.

Fig. S1. Growth performance, gastrointestinal morphology and SCFAs of goat kids at different developing ages.

Fig. S2. Generation of the ruminant GIT microbial catalog (RGMC) from seven public available large cohort studies in ruminants and Hungate collection genomes.

Fig. S3. Percentage increase of the proportion of reads, partitioned by GIT region or developmental age by GKGMC (goat kid GIT microbial catalog) in in relation to RGMC and GMMC (goat multi-kingdom microbial catalog).

Fig. S4. Distribution of microorganisms at phylum-level among four microbial genome catalogs (RGMC, GMMC, GFMC and GKGMC in this study). GFMC, goat fecal microbial catalog.

Fig. S5. Distribution of microorganisms at genus-level among four microbial genome catalogs (RGMC, GMMC, GFMC and GKGMC in this study).

Fig. S6. Functional annotation of GKGMC against KEGG, eggNOG and CAZy databases.

Fig. S7. Distribution of different classes of metabolic gene clusters (MGCs) across dominant phyla in GKGMC based on gutSMASH.

Fig. S8. PCoA analysis of GIT effect on microbial composition and function based on bray-curtis dissimilarity.

Fig. S9. Co-occurrence interaction network of the 1,002 MAGs.

Fig. S10. Analysis of microbial assembly dynamics using iCAMP.

Fig. S11. Changes in microbial carbohydrate-related functional maturation in the GIT microbiota of goat kids from birth to rumination.

Additional file 3. Supplemental Tables 1 to 12.

Table S1. Detailed information of 60 goat kids used in this study. **Table S2.** Experiment design of 124 samples in this study. **Table S3.** Summary of sequence data generated from GIT samples of 124 goats. **Table S4.** Genomic statistics for 1002 high quality nonredundant MAGs from our dataset. **Table S5.** Published ruminant metagenomic MAGs datasets used in this study. **Table S6.** GTDB classification for 1002 high quality non-redundant MAGs from our dataset. **Table S7.** Microbial phylum-level composition of four datasets (RGMC, GMMC, GFMC, and GKGMC) based on GTDB. **Table S8.** Microbial genus-level composition of four datasets (RGMC, GMMC, GFMC and GKGMC) based on GTDB. **Table S9.** The reference genome sequences for phylogenetic analysis from NCBI database. **Table S10.** Functional categories of cellulase, hemicellulase, amylase, and pectinase. **Table S11.** Gene number of CAZyme at family level of each MAG. **Table S12.** Detail of PULs in each MAG.

Additional file 4. Supplemental Tables 13 to 19.

Table S13. KOs for SCFA production. **Table S14.** Phylogenetic analysis of genes involved in SCFA production and their expression profile in GIT. **Table S15.** KOs for PTS system. **Table S16.** Phylogenetic analysis of genes involved in PTS system and their expression profile in GIT. **Table S17.** KOs for glycolysis. **Table S18.** KOs for methanogenesis. **Table S19.** KOs for terminal reductases.

Additional file 5. Supplemental Tables 20 to 22.

Table S20. DESeq2 results of differential MAGs among phytobiotics vs. control, antibiotics vs. control. **Table S21.** CAZyme gene number of each differential MAG. **Table S22.** KO gene number of each differential MAG.
